# Phytocomplex Characterization and Biological Evaluation of Powdered Fruits and Leaves from *Elaeagnus angustifolia*

**DOI:** 10.3390/molecules25092021

**Published:** 2020-04-26

**Authors:** Simone Carradori, Francesco Cairone, Stefania Garzoli, Giancarlo Fabrizi, Antonia Iazzetti, Anna Maria Giusti, Luigi Menghini, Sengul Uysal, Gunes Ak, Gokhan Zengin, Stefania Cesa

**Affiliations:** 1 Department of Pharmacy, University “G. d’Annunzio” of Chieti-Pescara, 66100 Chieti, Italy; luigi.menghini@unich.it; 2 Department of Drug Chemistry and Technologies, “Sapienza” University of Rome, 00185 Rome, Italy; francesco.cairone@uniroma1.it (F.C.); stefania.garzoli@uniroma1.it (S.G.); giancarlo.fabrizi@uniroma1.it (G.F.); antonia.iazzetti@uniroma1.it (A.I.); 3 Department of Experimental Medicine, “Sapienza” University of Rome, 00185 Rome, Italy; annamaria.giusti@uniroma1.it; 4 Erciyes University Halil Bayraktar Health Services Vocational College, Kayseri 38039, Turkey; sennguluysal@gmail.com; 5 Ziya Eren Drug Application and Research Center, Erciyes University, Kayseri 38039, Turkey; 6 Department of Biology, Science Faculty, Selcuk University, Konya 42130, Turkey; akguneselcuk@gmail.com (G.A.); gokhanzengin@selcuk.edu.tr (G.Z.)

**Keywords:** *Elaeagnus angustifolia*, MW-assisted extraction, scCO_2_-assisted extraction, pigments, polyphenols, HS-GC/MS, HPLC-DAD, antioxidant activity, enzyme inhibition activity

## Abstract

Fully ripe fruits and mature leaves of *Elaeagnus angustifolia* were harvested and analyzed by means of analytical and biological tests to better comprehend the chemical composition and therapeutic/nutraceutical potential of this plant. Fruits and leaves were dried and the obtained powders were analyzed to study their color character and (via headspace gas chromatography) describe the chemical profile. Subsequently, they were submitted to a chloroform–methanol extraction, to a hydroalcoholic extraction procedure assisted or not by microwaves, and to an extraction with supercritical CO_2_, assisted or not by ethanol as the co-solvent, to detect the polyphenolic and the volatile content. The resulting extracts were evaluated in terms of chlorophyll and carotenoid content, polyphenolic content, volatile fraction, total phenolic content, total flavonoid content, antioxidant activity, radical scavenging activity, and enzymatic inhibition activity. The results confirmed the correlation between the chemical composition and the high antioxidant potential of leaf extracts compared to the fruit extracts in terms of the phenolic and pigment content. A promising effect against tyrosinase emerged for all the extracts, suggesting a therapeutic/nutraceutical use for this plant. Conversely, the volatile content from both natural matrices was similar.

## 1. Introduction

*Elaeagnus angustifolia* L., also known as the oleaster or Russian olive, is a riparian bush native to Southern Europe and Western Asia and belongs to the Elaeagnaceae family, which comprises about 50 species. Used as an ornamental shrub as well as a soil stabilizer, but also regarded as an invasive species in Western North America, it is a highly drought- and cold-resistant plant, characterized by reddish sweet and sour berries [1]. The edible fruits, consumed fresh or dried, are a rich source of vitamins such as tocopherol, vitamin C, B1, and α-carotene, as well as minerals (potassium, sodium, and phosphorous) [2], and have traditionally been used in folk medicine for their analgesic, antipyretic, and diuretic activities, whereas the seeds are used for the extraction of an oil rich in polyphenols and other antioxidant molecules.

As reported in the literature [3], many interesting compounds, such as flavonoids, vitamins, minerals, sugars, sterols, and alkaloids, detected in aqueous and organic extracts obtained from the berries, could justify their use as an ingredient in food supplements or drug formulations. In fact, due to this rich phytocomplex, antimicrobial, insecticidal, antiarthritic, anti-inflammatory, cardioprotective, hypolipidemic, antimutagenic, antitumor, antioxidant, and gastroprotective effects are well recognized [4].

The proven relationship between oxidative stress and human diseases had led to increasing interest in polyphenol- and flavonoid-containing extracts, such as those derived from *Elaeagnus angustifolia* leaves and fruits. Literature data reported the presence in oleaster fruits and pulp of phenolic acids, mainly represented by *p*-hydroxybenzoic, caffeic and protocatechuic acid [5], differently glycosylated isorhamnetin, quercetin and kaempferol derivatives, and catechins [3,4]; the total phenolic and flavonoid contents of leaves and flowers were also evaluated, showing higher contents in the ethanol extracts from leaves [6]. Moreover, anticancer properties and radical scavenging activity are attributed to flavonoids and pro anthocyanosides extracted from *Elaeagnus angustifolia* [3].

Bioactive alkaloids were also detected in oleasters, such as eleagnin and calligonin, tetrahydroharmans that have a recognized inhibitory effect on monoamine oxidase [7], as well as homeostatic effects on blood pressure and antimalarial activity. These compounds are mainly present in the roots, bark, and aerial parts of the plant [3]. In reference to the essential oil, obtained by steam distillation of flowers or leaves, the chemical composition was recently investigated by GC-MS and GC-FID analyses and the radical scavenging by 2,2-diphenyl-1-picrylhydrazyl (DPPH) test; the toxicity against brine shrimp was also assessed. More than 50 and 25 compounds were extracted from flowers and leaves, respectively, among which cinnamates, farnesyl derivatives, and benzoates were detected [8].

*E. angustifolia* leaf and fruit extracts, traditionally employed for treating common illnesses, were recently re-evaluated by several scientific reports about their efficacy and their relationship with the content of bioactive compounds [2]. The anti-ulcer activity of methanolic extracts obtained from the whole fresh fruit was tested with relevant results. A significant gastroprotective effect was also demonstrated for the separated carotenoid fraction from the fruit seed oil [9]. Aqueous extracts obtained from the dry powdered fruit were tested for their antinociceptive and anti-inflammatory activity, showing promising effects on chronic pain and inflammation by inhibition of the cyclooxygenase type 2 enzyme [10]. Conversely, the muscle-relaxant effect (in a dose-dependent manner) and antitumor activity exerted by *Elaeagnus* fruits (as tested on HeLa cells) were attributed to the flavonoid content of seed aqueous and ethanolic extracts [11] and of flesh ethyl acetate extracts, respectively.

The antioxidant and antiradical activities of seven genotypes of oleaster methanol extracts were recently evaluated [12] by radical scavenging assays and by total phenolic and flavonoid content. The authors concluded that significant differences existed among the genotypes and that fruit seeds showed better antioxidant activity and higher phenolic contents with respect to the flesh and peels.

A recent review summarized the efficacy of *E. angustifolia* whole fruit aqueous extract on an osteoarthritis model, relating the anti-inflammatory effects via the inhibition of TNF-α, COX-1, COX-2, and IL-1β [13]. Due to its anti-inflammatory activity, the hydroalcoholic extract of this plant was also tested as a wound-healing agent in oral mucositis. Oral mucositis, which represents a frequent chemotherapy side effect affecting the quality of life of patients during anticancer therapy, could be significantly reduced by a daily application of the tested extract [14]. Hydroalcoholic extracts were also successfully tested on an experimental model of ovariectomy-induced osteoporosis in rats [15]. Previous studies have shown that estrogen deficiency provoked oxidative stress reducing bone antioxidant defenses and induced IL-6, TNF-α, and IL-1. The role of pro-inflammatory cytokines was also highlighted. Therefore, a synergic effect of the phytosterols detected in the *Elaeagnu*s extracts, in particular β-sitosterol and stigmasterol, with the anti-inflammatory polyphenols, could be taken into consideration for osteoporosis prevention. A significant chemopreventive effect of oleaster fruit against primary liver cancer induced in rats by diethylnitrosamine was revealed by Amereh et al. [16]. GSH levels were significantly increased and lipid peroxidation lowered, according to the hepatoprotective activity mediated by the antioxidant, anti-inflammatory, and antimutagenic mechanisms.

Moreover, *Elaeagnus* fruit and leaf extracts were both tested as antimicrobial agents on antibiotic-resistant microorganisms, showing good activity towards *Escherichia coli* and *Salmonella typhimurium* [17,18]. Finally, *Elaeagnus* polysaccharides were investigated for the best extraction conditions and their physicochemical and functional properties, and have been shown to exert anti-diabetic, antitumor, and immunological effects [19]. In another report, two polysaccharide components from the fruit pulp were isolated, characterized for their average molecular weight and monosaccharide composition, and evaluated in relation to their antioxidant and free radical scavenging activity [20]. Three crude polysaccharides tested for their immunological effects induced an increase in NO release and enhanced the macrophages’ phagocytic activity [21], implying a potential use of oleaster as a functional food.

On the basis of prior knowledge, the present work aimed to compare the effect of different extraction techniques, performed on dried whole fruits and leaves of *Elaeagnus angustifolia,* in relation to the chemical composition of the obtained products. First, the leaves’ and fruits’ dry powders were submitted in their solid form to HS-GC/MS and CIEL*a*b* colorimetric analysis. Then, all the different obtained extracts were analyzed by colorimetric analyses and further studied for their chemical composition by HPLC-DAD, GC/MS, and ^1^H and ^13^C NMR analysis. Total carotenoid, chlorophyll, polyphenolic, and flavonoid contents were evaluated by UV-VIS spectroscopy. The extracts were also tested for some interesting biological activities, such as total antioxidant capacity, free radical scavenging activity, and enzyme inhibition activity.

## 2. Results and Discussion

### 2.1. Extraction Methods

**Figure 1:**
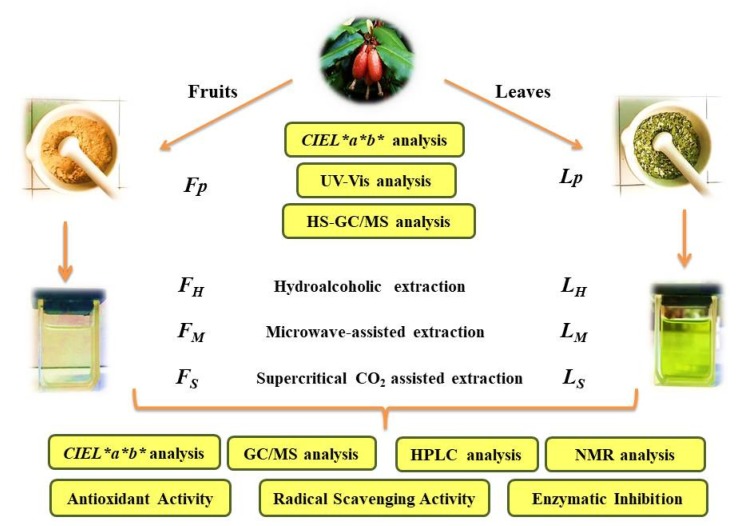
Flowchart of the adopted work-up.

The present work aimed to compare the effect of different extraction techniques, performed on dried whole fruits (*F* series) and leaves (*L* series) of *Elaeagnus angustifolia*, in relation to the chemical composition of the obtained extracts. (Figure 1)

All extraction experiments were performed in triplicate (error falls in the range of 5%–8%, data not shown). In the *F* series, mean extraction yield values range between the very low value of 0.8% (g extract/100 g powder) in *F_S_* and the very high yield of 61.2% in *F_H_*. Indeed, in the *L* series, mean extraction yield values fall in a narrower range, between a low 1.2% in *L_S_* and a 15.0% yield in *L_H_*. The highest value found in *F_H_* is probably due to a co-extraction of sugars rather than to a higher polyphenolic content, as shown by the HPLC data analysis. The lowest values were observed in *F_S_* and *L_S_*, due to the fact that SCE probably allows a very selective extraction of fruit and leaf components, thus representing a valuable method to extract specific molecules, besides providing shorter extraction times and a green procedure [22]. The addition of co-solvents modifies the system solubility and helps to increase extraction yields [23]. In this case, the use of the co-solvent ethanol for the *Elaeagnus* powdered leaves increased the extraction yield, allowing for the extraction of more polar molecules.

The use of high temperatures in the SCE technique could cause a partial degradation of the fruits’ sugar content, mainly represented by fructose and glucose [5], into compounds such as 5-hydroxymethylfurfural, furfural, and other furanyl derivatives [24], which were found at the end of this process as isolated compounds. Different furanyl derivatives were also detected by the HS-GC-MS in *Fp*, and in its extracts *F_H_* and *F_M_* and even, in small quantity, in *Lp*. In the *Fp* and *Lp* samples, they could simply be the result of the dehydration process.

Besides the compositional divergences among extracts, further demonstrated by the performed analyses, some differences were also shown in terms of yields: when HAE and MAE methods were applied using the same extraction solvent type, solid-to-liquid ratio and pressure, the resulting MAE (*F_M_*: 48.0% and *L_M_*: 6.7%) was less efficient than HAE, probably due to the better selectivity in polyphenolic extraction [25] (these data will be further confirmed by HPLC data analysis). Considering that phenolics and carotenoids can be very sensitive to oxidation, especially if they are held at high temperature for prolonged times, MAE represents a valid alternative extraction method to guarantee safer recovery of these bioactive compounds, as well as to maintain their antioxidant potential and avoid matrix degradation, furnishing reproducible, robust, and fast analytical procedures.

### 2.2. Color Analysis

The color of a foodstuff is not only correlated to its complex chemical profile, and particularly to the pigment content of a certain matrix, but also depends on the procedures to which a specific raw material is submitted during its entire processing cycle, from harvesting to the shelf [26].

**Figure 2:**
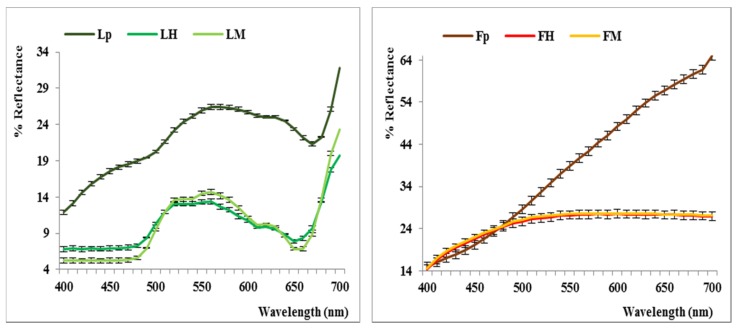
Reflectance curves of *E. angustifolia* fruits and leaves (powders and extracts).

Powdered fruits and leaves (*F_P_* and *L_P_*) of *E. angustifolia*, obtained by dehydration and stored sealed, were opened immediately before the colorimetric analyses. The CIEL*a*b* parameters reported in Table 1 and the reflectance curves shown in Figure 2 correspond to the pale yellow color of the fruit and the grayish-green color of the leaves. Each experiment is reported as the mean of four measurements and the results are expressed as the mean value ± standard deviation.

To the best of our knowledge, tristimulus colorimetry has never been applied to *Elaeagnus* samples and it is quite difficult to give an interpretation of these data as they are influenced by many different parameters [27]. At any rate, the positive a* value (7.73, in the red region), combined with the high b* value (27.23 in the yellow region) of the dehydrated fruits, could account for a weak carotenoid and a more significant flavonoid pigment contribution, whereas the negative a* (–1.47, green region) combined with a lower b* value (13.14, yellow region) could account for the residue chlorophyll content of the powdered leaves. A higher luminance was expressed by the fruit powder (L* = 68.61) compared with the value expressed by the leaf powder (L* = 56.29), as shown by the darker chlorophyll pigment.

Subsequently, the hydroalcoholic extracts obtained from fruits and leaves, assisted (*F_M_* and *L_M_*) or not (*F_H_* and *L_H_*) by microwaves, were also submitted to colorimetric analyses in the same conditions previously adopted for the powdered samples. Reported data show lower L* values with respect to the powdered samples (59 vs. 68 for the *F_H_* series and 41 vs. 56 in the *L_H_* series) and negative or more negative values of a* (−2 vs. 8 for the *F_H_* series; −8 vs. −1.5 in the *L_H_* series); regarding the yellow parameter, much lower values of b* (8.5 vs. 27) in the *F_H_* series and different behavior for *L_H_* and *L_M_* with respect to *Lp* were shown (14.41 and 22.41 vs. 13.14). High differences were finally revealed in terms of standard deviation in the samples extracted by MAE.

On the whole, darker samples were obtained after both hydroalcoholic extractions, probably in agreement with the higher expression of the colored fraction in the liquid form. In any case, it is difficult to draw conclusions from the comparison of such different samples (liquid vs. solid), considering the multiple effects of powdered samples’ surfaces on the final (expressed and perceived) color. As shown in Figure 2, the reflectance curves related to the *F_H_*–*F_M_* and *L_H_*–*L_M_* extraction processes largely overlap. This suggests the presence of a similar polyphenolic profile from a qualitative point of view, but not necessarily the presence of a similar content in quantitative terms, as subsequently shown by the HPLC-DAD data.

### 2.3. Carotenoid and Chlorophyll Analysis

*Elaeagnus angustifolia* leaves showed a 45% higher concentration of chlorophyll a than chlorophyll b (*p* < 0.002), and a total carotenoid concentration 60% lower than chlorophyll a (18.3 μg/g and 43.8 μg/g respectively, Table 2). The chlorophyll a/b ratio was 1.8, demonstrating that examined leaves come from shade plants that possess much higher amounts of LHC-II (Light Harvesting Complex) than sun-exposed plants. A similar chlorophyll a/b ratio was also found in *Thymus algeriensis* [28]. Nevertheless, the ratio of Chls a and b to total carotenoids showed a value of 3.7, typical of leaves that become more yellowish green, compared to dark green leaves that exhibit, on average, a ratio of 4.5.

Conversely, in *Elaeagnus* fruits, chlorophyll b had almost double the concentration of chlorophyll a and the levels of total carotenoids were 3:1 with respect to chlorophyll b and about 70% higher than chlorophyll a, respectively (Table 2). In fact, the chlorophyll a/b ratio is very low (0.53), indicating a high level of chlorophyll b. Furthermore, the very low value of Chls a and b to the total carotenoid ratio (0.91) would indicate the fruit’s color change (probably due to ripening) from green to yellow-orange, during which this ratio decreases below 1.0.

### 2.4. HS-GC/MS and GC/MS Results

Headspace/Gas Chromatography–Mass Spectrometry (HS/GC-MS) analyses of dried leaves and fruits, before the extraction process, led to the identification and quantification of 32 compounds (Table 3).

The main component was furfural (36.7% in fruits; 18.8% in leaves) followed by 5-methylfurfural, (25.3% in fruits; 9.6% in leaves). This and other found furfural derivatives could come from the partial dehydration of the fruit content of sugars and even of the pentose sugars of the leaf cell wall, occurring during the air-drying procedure. Some minor compounds present in percentages higher than 2%, including dihydro-2-methyl-furanone (5.8%), 4-cyclopentene-1,3-dione (4.5%), and 5-hydroxymethylfurfural (2.7%), were found only in the fruits. On the other hand, in the leaves some compounds were found that were not identified in the fruits. These include *p*-vinylguiacol (9.3%), pyrrolidine, 2-(cyanomethylene) (8.1%), pyrimidine, 2-methyl (7.2%), 1*H*-pyrrole-2-carboxaldehyde (6.4%), and *p*-xylene (5.3%).

After the extractions, HAE and MAE samples were examined by GC-MS. All results are shown in Table 4.

In total, 13 compounds were identified. Furfural (28.7%) and furaneol (16.9%) in fruits, and *p*-vinylguaiacol (48.0%) and acetol (20.4%) in leaves, were the most abundant components after MAE. A similar chemical profile was found for HAE leaves, where *p*-vinylguaiacol (39.5%) and acetol (24.0%) remained the principal components. On the contrary, a different trend was highlighted for *F_H_* in which 5-hydroxymethylfurfural (21.6%) and acetol (18.6%) were the principal components (see Section 3.6). The same nine components in MAE and HAE fruits were revealed, but, for four of them, significant differences in the percentages could be highlighted (MAE vs. HAE: furfural 28.7% vs. 2.3%; 2-furanmethanol 7.7% vs. 18.2%; 2-pyranone 10.9% vs. 17.3%; 5-hydroxymethylfurfural 11.3% vs. 21.6%). Kim and Chung reported the presence of furan compounds in dried *Lycium chinensis* Miller fruit extracts [29]. A lower number of components, five in total, was identified in MAE and HAE leaves. Among these, only the main component *p*-vinylguaiacol showed significant differences (MAE vs. HAE: 48.3% vs. 39.5%). Acetol was the only compound that was found in all four extracts, with a higher percentage in the leaves than in the fruits (20.4% and 24.0% vs. 6.4% and 18.6%).

The expected significant differences, already found in the headspace chemical profile and confirmed by GC-MS of the extracts, could account for the differences in the tested bioactivities. Mass spectra of identified compounds with a relative percentage higher than 10% were reported in the Supplementary Materials.

### 2.5. HPLC-DAD Analysis

**Figure 3:**
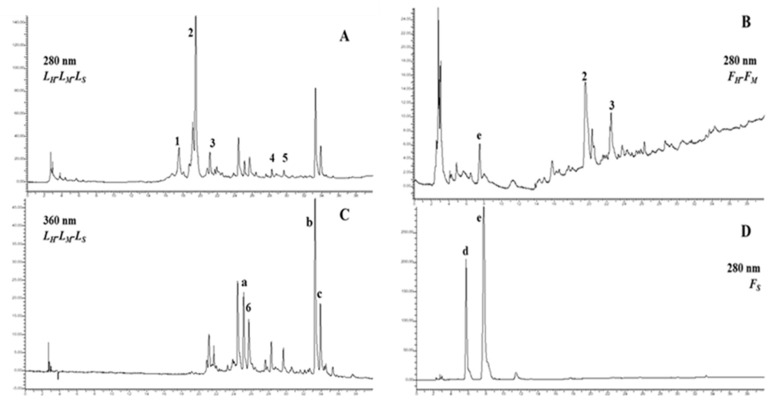
Chromatograms of phenolic compounds in leaf and fruit extracts by HPLC-DAD analysis at 280 (**A**,**B**,**D**) and 360 nm (**C**). Identified peaks: **1**. epicatechin, **2**. chlorogenic acid, **3**. caffeic acid, **4**. *p*-coumaric acid, **5**. ferulic acid, **6**. quercetin-3-D-galactoside; **a**. rutin, **b**. kaempferol glucoside derivative, and **c**. kaempferol were tentatively identified by literature [30,31].

The obtained extracts were then further characterized by HPLC-DAD analyses at 280 nm for the identification of the hydroxycinnamic profile and at 360 nm for the identification of the flavonoid profile. Examples of chromatographic profiles are shown in Figure 3.

The quantitative data of performed analysis are reported in Table 5, where the mean values are compared. The phenolic content of the analyzed samples (*L_H_*, *L_M_*, *L_S_* and *F_H_, F_M_*, *F_S_*), monitored at 280 nm, was quantified through the use of calibration curves that allowed for the identification of the epicatechin (**1**), chlorogenic acid (**2**), caffeic acid (**3**), *p*-coumaric acid (**4**), and ferulic acid (**5**), whereas the sum of the peak areas of flavonoid compounds, monitored at 360 nm, was expressed as quercetin-3-D-galactoside (**6**) equivalents. As shown in Figure 3, if in the *L* series (panel A) the polyphenolic profile is similar from a qualitative point of view, in the *F* series a different chromatographic pattern is observed in *F_S_* (panel D). The *F* series at 360 nm was not reported because the flavonoid profile was not detected. The chromatograms at 280 nm illustrate the typical hydroxycinnamic pattern of *Elaeagnus* samples, in which chlorogenic acid is the main component in both *L* and *F* series, followed by epicatechin, caffeic acid, *p*-coumaric acid, and ferulic acid. The retention times of these molecules were confirmed both by analysis conducted on the pure reference standards and by data reported in the literature [30]. In particular, as shown by Table 5 in *F_H_* and *F_M_*, it was only possible to identify chlorogenic and caffeic acid, whose amount is much lower than in the respective *L_H_* and L*_M_* (chlorogenic acid content*: F_H_* vs *L_H_*, 1.1 vs 40.8 mg/g dry extract; *F_M_* vs *L_M_*, 3.5 vs 41.9 mg/g dry extract; caffeic acid content: *F_H_* vs *L_H_*, 0.19 vs 2.4 mg/g dry extract; *F_M_* vs *L_M_*, 0.4 vs 3.18 mg/g dry extract). Moreover, the hydroxycinnamic content found in *F_M_* was about 3-fold that of *F_H_*, whereas there were not substantial differences in the amount of chlorogenic acid, caffeic acid, *p*-coumaric acid, and ferulic acid between the two applied hydroalcoholic extractions (*L_H_*-*L_M_*). Indeed, the epicatechin content found in *L_M_* was about 3-fold higher than in *L_H_* (43.10 vs 13.30 mg/g dry extract). Instead, important differences can be observed in *L_S_* as compared to *L_H_*–*L_M_*, in which the chlorogenic acid content (4.85 mg/g dry extract) was about one-tenth that of the other two analyzed extracts (40.8 and 41.9 mg/g dry extract, respectively).

The chromatograms monitored at 360 nm (panel C) show the flavonoid profile of analyzed samples. Quercetin-3-D-galactoside (**6**) was the only peak identified by analysis on the pure reference standard, whereas the other peaks (**a**–**c**) were tentatively identified from the literature [32,33] as rutin, kaempferol, and a kaempferol glucoside derivative, respectively. Kaempferol was confirmed as the main flavonoid component of *E. angustifolia* [31]. The flavonoid content was particularly represented in samples *L_H_* and *L_M_* (5.40 and 6.79 mg/g dry extract, respectively). The flavonoid content in *L_S_* was, also in this case, about 8-fold lower than that in *L_H_* and *L_M_* (0.80 mg/g dry extract). The obtained data are comparable with the literature, where only the amount of kaempferol reaches values between 0.02 and 2.00 mg/g of leaf extract [34].

The *F_S_* sample (panel D) shows a completely different chromatographic profile, characterized by the presence of only two peaks (**d** and **e**) not correlated to polyphenolic molecules. For this reason, this extract was subjected to ^1^H NMR and ^13^C NMR analysis and to a purification step for further identification of the contained molecules (Section 3.6).

In conclusion, there are not substantial differences in the polyphenolic profile between the two hydroalcoholic extraction methods, either in the *F* series or in the *L* series (as previously stated in the colorimetric evaluation), whereas significant differences can be observed in the extracts deriving from SCE. The results obtained by the HPLC-DAD analysis lay the foundation for the evaluation of the antioxidant properties of the differently obtained extracts.

### 2.6. H- and ^13^C-NMR Analysis

The *F_S_* sample, in which the presence of two unidentified molecules was shown by HPLC-DAD analysis, was analyzed by NMR spectroscopy. The ^1^H NMR analysis confirmed the presence of two compounds that, once purified by semipreparative HPLC and further characterized by NMR analysis, were identified as 5-(hydroxymethyl)furfural (39%) and 4,5,6-trihydroxyhex-3-en-2-one (61%).

The results confirmed the molecular formula. 5-(Hydroxymethyl)furfural spectra (^1^H and ^13^C NMR) were recorded in DMSO-*d*_6_ as the solvent and shown in the Supplementary Materials. Splitting patterns are designated as b (broad), s (singlet), d (doublet), t (triplet), q (quartet), or m (multiplet). The value of chemical shifts has been discussed according to [35] and reported below (pale yellow oil; ≈ 20 mg; ^1^H NMR (400.13 MHz) (DMSO-*d*_6_): *δ* (ppm) 9.55 (s; 1H), 7.50 (d; *J* = 3.5 Hz; 1H), 6.61 (d; *J* = 3.5 Hz; 1H), 5.56 (t; *J* = 5.9 Hz; 1H), 4.50 (d; *J* = 5.9 Hz; 2H); ^13^C NMR (100.6 MHz) (DMSO-*d*_6_): *δ* 178.4; 162.6; 152.2; 124.9; 110.1; 56.4. These results agree with the reported data observed by HS-MS and GC-MS analysis, where furfural was the main volatile compound present in fruit.

4,5,6-Trihydroxyhex-3-en-2-one spectra (^1^H and ^13^C NMR) were recorded in DMSO-*d*_6_ as the solvent and shown in the Supplementary Materials. The value of chemical shifts has been discussed and reported below (colorless oil, ≈ 15 mg; ^1^H NMR (400.13 MHz) (DMSO-*d*_6_): *δ* (ppm) 7.52 (s, 1H), 5.80 (bd, *J* = 4.2 Hz, 1H), 4.25 (dd, *J_1_* = 9.5 Hz, *J*_2_ = 8.4 Hz, 1H), 4.07-3.99 (m, 2H), 1.90 (s, 3H); ^13^C NMR (100.6 MHz) (DMSO-*d*_6_): *δ* 187.8, 158.2, 131.8, 71.8, 67.8, 15.8.

### 2.7. TPC, TFC, and Antioxidant In Vitro Assays

The analysis of the total bioactive components, along with the data reported in Section 3.3, revealed that the extracts, regardless of the origin (fruit or leaves), presented a different chemical profile (Table 6). In more detail, the extracts from leaves were characterized by much higher values of TPC (17.86 ± 0.70–65.35 ± 0.52 mg GAE/g) and TFC (24.34 ± 0.24–36.58 ± 0.35 mg RE/g) than the fruit extracts (3.32 ± 0.04–5.15 ± 0.03 mg GAE/g and 0.17 ± 0.02–0.22 ± 0.02 mg RE/g, respectively), as also evidenced by our HPLC studies. These results were also better than those obtained using only ethanol or methanol as the extracting solvent, as reported by Saboonchian et al. [6]. Collectively, for leaves and fruits, a general trend can be extrapolated: hydroalcoholic maceration is comparable to microwave-assisted extraction but gives different results than SCE. Due to the presence and amount of these classes of compounds, well known to exert a beneficial effect on the health status of the consumer/patient [36], we evaluated the ability to counteract oxidative stress by means of different in vitro spectrophotometric assays.

As regards the antioxidant and chelating activities, all the extracts from fruits displayed inferior values to those from leaves, probably due to the presence of oxygenated and low-molecular-weight compounds (alcohols, aldehydes), as also reported by Torbati et al. [8]. The fruit extracts were almost comparable in terms of results, whereas the leaf extracts followed the trend *L*_H_ ≈ *L_M_* ˃˃ *L_S_*. The use of six different and well-recognized in vitro assays led to a better comprehension of the antioxidant mechanisms (electron transfer ability, radical stabilization, total antioxidant activity, and metal chelation) characterizing these extracts.

### 2.8. Enzyme Inhibition Assays In Vitro

Due to the particular composition and amounts of important metabolites in the obtained extracts, we aimed to evaluate their ability to inhibit crucial enzymes for the treatment of human diseases (e.g., type 2 diabetes and Alzheimer’s disease). Indeed, a plethora of studies demonstrated that chlorogenic acid [37] and flavonoids [32] can be useful for the treatment of Alzheimer’s disease as multitarget agents—not only acting as radical scavengers [33], but also inhibiting key enzymes (cholinesterases) involved in the pathophysiology of this neurological disorder. Moreover, chlorogenic acid, epicatechin, rutin, ferulic acid, *p*-coumaric acid, and caffeic acid were shown to inhibit tyrosinase [38,39,40]. Finally, phenols and flavonoids can be used in the treatment of diabetic complications, reducing vascular inflammation and oxidation [41,42].

Thus, we were prompted to test our extracts, in which we detected these bioactive compounds as well as the nonpolar compounds by GC/MS, on a panel of enzymes involved in those pathologies. As reported in Table 7, fruit and leaf extracts had no or weak inhibitory activity against two enzymes involved in the regulation of the intestinal monosaccharides’ absorption (α-amylase and α-glucosidase), whereas they showed a promising inhibition of both cholinesterases (AChE and BChE). The results among the different extracts were almost interchangeable, except for those obtained for *L_S_*, which displayed a better inhibitory activity against butyrylcholinesterase. In addition, the best tyrosinase inhibitory effect was observed for *L_H_* with a value of 61.20 mg KAE/g, followed by *L_M_* and *L_S_*. The results indicated that *E. angustifolia* had significant potential as a source of natural enzyme inhibitors, especially against tyrosinase.

## 3. Materials and Methods

### 3.1. Materials

*Elaeagnus angustifolia* leaves and fruits were collected from wild plants from Turkey (Konya) and were botanically and morphologically identified by Dr. Evren Yıldıztugay from the science faculty of Selcuk University, Konya, Turkey. Fruits were in a ready-to-eat ripeness state.

Double-distilled water, ethanol, 98% formic acid, acetonitrile RS, *n*-hexane for HPLC, and dichloromethane were purchased from Merck Life Sciences s.r.l (Milan, Italy), methanol for HPLC and diethyl ether were purchased from Carlo Erba Reagents (Milan, Italy), DMSO-*d*_6_ (99.80% D) was obtained from Eurisotop (Saint-Aubin, France) and CO_2_ was purchased from Sapio s.r.l. (Monza, Italy).

### 3.2. Sample Preparation

Whole fruits or mature long elliptic leaves were washed and then straightaway submitted to a dehydration process. The samples were distributed over a steel grid and dried in a dedicated box, avoiding light exposure. Forced ventilation at room temperature (25 °C) was applied until a constant weight was reached. After 10 days, the weight loss on drying was determined to be <10%, as recommended by the European Pharmacopeia guidelines. A laboratory mill (Retsch Cutting Mill SM 200, Haan, Germany) was used to grind the samples (powder size: about 1 mm). The powders (fruit powder, *Fp*; leaf powder, *Lp*) were stored in sealed bags in the dark at 4 °C until they were directly analyzed or subjected to the different extraction procedures (Figure 1).

### 3.3. Extraction Methods

#### 3.3.1. Hydro-Alcoholic Extraction (HAE) by Maceration

About 230 mg of the samples were extracted with 10 mL ethanol:water in 70:30 (*v:v*) ratio by stirring for 1 h at room temperature in the dark (*F_H_* and *L_H_*). The extraction mixture was decanted, filtered on paper, and the hydroalcoholic solvent was evaporated at 40 °C in the dark under a vacuum and immediately analyzed or stored at 4 °C.

#### 3.3.2. Microwave-Assisted Extraction (MAE)

MAE was performed using an automatic Biotage Initiator^TM^ 2.0 (Uppsala, Sweden) characterized by 2.45-GHz high-frequency microwaves and a power range of 0–300 W. The internal vial temperature was strictly monitored by the infrared (IR) sensor probe. About 230 mg of the samples were transferred to a sealed 10 mL vessel suitable for an automatic single-mode microwave reactor and 10 mL of ethanol:water in a 70:30 (*v*:*v*) ratio were added to the sample. MAE was carried out by microwave irradiation for 7.3 min at 55 °C (corresponding to 1 h maceration at room temperature, following the Arrhenius equation and with the same solid-to-liquid ratio used in Section 3.3.1). The extraction mixture was decanted, filtered on paper, evaporated at 40 °C in the dark under vacuum, and immediately analyzed or stored at 4 °C (*F_M_* and *L_M_*).

#### 3.3.3. Supercritical CO_2_ Assisted Extraction (SCE)

SCE was carried out in a laboratory-scale supercritical fluid extraction system (Jasco Europe Srl, Cremella, Italy). The system was operated in a semi-continuous mode by pumping scCO_2_ with a Jasco-PU1580 unit, through a stainless-steel column, 10 mm i.d. and 200 mm length, equipped with 10 µ porous stainless steel 316 L sintered filter disks. The column was filled with the dehydrated samples, heated using a Jasco CO-4061 oven to the chosen temperature, and scCO_2_ was pumped to the target pressure. After a pre-equilibrating time of 30 min, SCE was performed.

The dried leaves (7.0 g) were transferred in the CO_2_ extractor added with 5 mL ethanol and extracted for 1 h at 150 bar and 55 °C. The dried fruits (7.0 g) were transferred to the CO_2_ extractor and extracted for 1 h at 250 bar and 80 °C. The extraction mixtures (*F_S_* and *L_S_*) were immediately analyzed by ^1^H and ^13^C NMR and HPLC analysis or stored at 4 °C until the analyses were performed.

### 3.4. Colorimetric Analysis

The powders and resulting dried extracts were analyzed for their color with an X-Rite SP-62 colorimeter (X-Rite Europe GmbH, Regensdorf, Switzerland), set with a D65 illuminant and a 10° observer angle, as previously described [43]. Each experiment was performed four times and the results are expressed as the mean value ± standard deviation (SD).

### 3.5. Carotenoid and Chlorophyll Analysis

The total carotenoid and chlorophyll (a and b) contents in *E. angustifolia* leaf and fruit samples (*F_P_* and *L_P_*) were detected as reported in [44].

In brief, 50 mg of each sample were separately homogenized with a mortar and pestle in 6 mL of chloroform–methanol (2:1, *v*/*v*) in the presence of MgO (20 mg). The obtained homogenates were filtered on paper and distilled water was added to the amount of 20% of the extract volume. Finally, the mixture was centrifuged and the phases separated. The absorption spectrum of the chloroform phase was recorded with a Beckman Coulter (Pasadena, CA, USA) DU 800 spectrophotometer, in the range 350–800 nm with a spectral resolution of 0.5 nm, at a temperature of 20 °C. Both chlorophylls and total carotenoids were determined using their absorption coefficient. Data are reported as the mean of three replicates and expressed as μg/g dw (dry weight) ± SD.

### 3.6. HS-GC/MS Analysis

To investigate the volatile fraction of dried samples, a PerkinElmer Headspace (HS) Turbomatrix 40 autosampler (Waltham, MA, USA) connected to a Clarus 500 GC-MS was used for headspace analysis [45,46]. To optimize the headspace procedure for the determination of volatile organic compounds (VOCs), parameters such as equilibration time and temperature were adjusted. The sampling procedure was performed as follows: fruit and leaf samples (14 mg and 120 mg, respectively) were put into a 20mL vial with 2 mL of diethyl ether and immediately tightly sealed with crimp aluminum caps and 20-mm white rubber septa (Merck KGaA, Darmstadt, Germany) using a vial crimper. The samples were incubated at 90 °C for 20 min, then the volume of headspace gas was transferred into the capillary column (Rtx-1) by a transfer line. The same temperature program, described in the next paragraph, was used for the GC/MS apparatus.

### 3.7. GC/MS Analyses

Gas chromatographic/mass spectrometric (GC/MS) analysis was carried out on *F_H_*, *F_M_* and *L_H_*, *L_M_* extracts, with both GC-MS and GC-FID, using a Turbomass Clarus 500 GC-MS/GC-FID from PerkinElmer Instruments [47,48]. A Stabilwax fused-silica capillary column (Restek, Bellefonte, PA, USA) (60 m × 0.25 mm, 0.25 μm film thickness) was used with helium as the carrier gas (1.0 mL/min). GC oven temperature was kept at 60 °C and programmed to 220 °C at a rate of 6 °C/min, and kept constant at 220 °C for 20 min. All mass spectra were recorded in the electron impact ionization (EI) at 70 eV. The mass range was 30–400 *m*/*z*. The *F_H_*, *F_M_* and *L_H_*, *L_M_* extracts (about 20 mg) were diluted in 1 mL of methanol and 2 μL of the obtained solutions were injected into the GC injector at a temperature of 280 °C. The identification of the main components was performed by comparison of their linear retention indices (LRIs) and spectral mass with those reported in the library data (Wiley 02 and Nist) of the GC/MS system. The LRI of each compound was calculated using a mixture of aliphatic hydrocarbons (C_8_-C_30_, Ultrasci, Bologna, Italy) injected directly into the GC injector with the same temperature program reported above. Relative abundances of the separated components were derived using the same instrumentation with the FID detector configuration, without the use of an internal standard or correction factors. Analyses were repeated twice.

### 3.8. HPLC-DAD Analyses

Dried extracts (*F_H_*, *F_M_*, *F_S_* and *L_H_*, *L_M_*, *L_S_*) were weighed, dissolved in methanol, and filtered before injection into an HPLC PerkinElmer apparatus consisting of a Series 200 LC pump, a Series 200 DAD, and a Series 200 autosampler, including a Totalchrom PerkinElmer software for the data acquisition. Chromatography was performed on a Luna RP18 column (250 × 4.6 mm i.d., 5 μm) using a mobile phase made by acetonitrile and water acidified by 5% formic acid, in a gradient with a flow rate of 1 mL/min, at 280 and 360 nm. Analyses were performed with a linear gradient from 98% acidified water to 50% acidified water over 33 min. Calibration curves were built and used for the quantitation of polyphenols, using at 280 nm epicatechin (*R^2^* = 0.9878), chlorogenic acid (*R^2^* = 0.9987), caffeic acid (*R^2^* = 0.9984), *p*-coumaric acid (*R^2^* = 0.9879), and ferulic acid (*R^2^* = 0.9974) and at 360 nm quercetin-3-d-galactoside (*R^2^* = 0.9999) as reference standards.

### 3.9. Semipreparative HPLC-Refractive Index Detector

Dried extract (*F_S_*) was weighed, dissolved in dichloromethane, and filtered before injection into an HPLC semipreparative apparatus, consisting of a Waters (Milford, MA, United States) Millipore 150 pump, a Gilson (Middleton, WI, USA) 132 refractive index detector, and Jasco (Easton, MD, United States) Borwin software for the data acquisition. Chromatography was performed on a Macherey-Nagel, (Bethlehem, PA, USA) 100-5 column. The eluent mixture used was 60/40 (*v*/*v*) *n*-hexane/ethyl acetate, and the flow rate was fixed at 5 mL/min. The analysis was performed at 25 °C.

### 3.10. H- and ^13^C-MR Analysis

^1^H and ^13^C NMR (400.13 and 100.03 MHz) analyses were recorded with a Bruker (Billerica, MA, United States)Avance 400 (Milano, Italy) spectrometer, equipped with a Nanobay console and Cryoprobe Prodigy Probe. Ten milligrams of *F_S_* sample were dissolved in 0.6 mL of DMSO-*d*_6_ (I.E% = 99.80%), transferred to a NMR tube, and analyzed. The resulting ^1^H NMR and ^13^C NMR spectra were processed using Bruker TOPSPIN software.

### 3.11. Determination of Total Bioactive Components: Total Phenolic Content (TPC) and Total Flavonoid Content (TFC)

The total phenolic content (TPC) was determined using the Folin–Ciocâlteu method according to Zengin et al. [49], calculated and expressed as gallic acid equivalent (GAE) in mg/g extract of plant material. In brief, 50 µL of extract (1 mg/mL) and 100 µL of Folin–Ciocâlteu reagent, diluted 1:9 in distilled water, were mixed. After 3 min, 75 µL of sodium carbonate (Na_2_CO_3_, 2% *w/v*) were added and then incubated at 25 °C in the dark for 120 min. The absorbances were measured at 765 nm.

The total flavonoid content (TFC) was calculated using a method previously described by Zengin et al. [28]. Rutin was used as the reference compound and the results were expressed as equivalents of rutin (mg RE/g). In brief, 200 µL sample solution was mixed with AlCl_3_ (2% in methanol). After 15 min of incubation, the absorbance of each sample was recorded at 420 nm.

### 3.12. Antioxidant and Metal Chelating Spectrophotometric Assays

The scavenging capacity of the free radical DPPH was monitored according to Zengin et al. [28]. In brief, 50 µL of the sample solution (1 mg/mL) were mixed with 0.004% methanolic solution (150 µL) of DPPH, a quite stable radical. The mixture was incubated for 30 min in the dark and the DPPH radical reduction was determined by measuring the absorption difference at 517 nm. Trolox was used as a standard reference and the DPPH results were expressed as Trolox equivalents per gram of dry extract (mg TE/g extract).

For the metal chelating activity [28], the test solution (100 µL, 1 mg/mL) was added to a FeCl_2_ solution (50 µL, 2 mM). The reaction was initiated by adding 5 mM ferrozine (100 µL) solution. Similarly, a blank was prepared for each sample without ferrozine. Then, the absorbance of the sample and blank was noted at 562 nm after 10 min incubation at room temperature. The results were expressed as milligrams of ethylenediamine tetracetic acid (EDTA) equivalents (E) per sample amount (mg EDTAE/g extract).

An ABTS (2,2′-azino-bis(3-Ethylbenzothiazoline-6-sulfonic acid) radical cation scavenging assay was carried out by generating the ABTS^+•^ radical cation obtained by the reaction of 7 mM ABTS solution with 2.45 mM potassium persulfate in darkness for 12–16 h. Prior to starting the assay, methanol was used to dilute the ABTS^+•^ solution to an absorbance of 0.700 ± 0.02 at 734 nm. The resulting ABTS^+•^ solution (25 µL) was mixed with the extract solution (200 µL) and the mixture was incubated for 30 min at room temperature. The absorbance was then measured at 734 nm. Trolox was used as a standard reference and the results were expressed as Trolox equivalents per gram of dry extract (mg TE/g extract) [28].

In FRAP (ferric ion reducing antioxidant power) assay, the reduction of Fe^3+^-TPTZ (2,4,6-tris(2-pyridinyl)-1,3,5-triazine) to blue-colored Fe^2+^-TPTZ complex was monitored by the method described by Zengin et al. [28]. It is a simple and straightforward test. Ten volumes of acetate buffer (300 mM, pH 3.6), one volume of TPTZ solution (10 mM TPTZ in 40 mM HCl), and one volume of FeCl_3_ solution (20 mM FeCl_3_·6 H_2_O in 40 mM HCl) were mixed to prepare the FRAP reagent. The reaction mixture (25 µL of sample and 200 µL of FRAP reagent) was incubated for 30 min in the dark at 25 °C. The absorbance was then measured at 734 nm. Trolox was used as a standard reference and the results were expressed as Trolox equivalents per g of dry extract (mg TE/g extract).

The cupric ion reducing activity (CUPRAC) was determined according to the method of Zengin et al. [28]. The sample solution (25 µL, 1 mg/mL) was added to a premixed reaction mixture (200 µL) containing CuCl_2_ (10 mM), neocuproine (7.5 mM) and NH_4_Ac buffer (1 M, pH 7.0). The sample absorbances were read at 450 nm after a 30-min incubation at room temperature. CUPRAC activity was expressed as milligrams of Trolox equivalents (mg TE/g extract).

The total antioxidant activity of the samples was evaluated by the phosphomolybdenum method according to Zengin et al. [28]. The sample solution (0.3 mL, 1 mg/mL) was added to 3 mL of reagent solution (0.6 M sulfuric acid, 28 mM sodium phosphate, and 4 mM ammonium molybdate). The sample absorbance was read at 695 nm after a 90-min incubation at 95 °C. The total antioxidant capacity was expressed as millimoles of Trolox equivalents (mmol TE/g extract).

### 3.13. Enzyme Inhibition Assays

α-Amylase inhibition was measured using a reaction mixture of 25 µL of extract solution (1 mg/mL) and 50 µL of *α*-amylase solution (10 U/mL) prepared in phosphate buffer containing 6 mM NaCl (pH 6.9). Following 10 min of incubation at 37 °C for 10 min, 50 µL of starch solution (0.05%) were added. To stop the reaction, 25 µL of HCl (1 M) were added. Subsequently, 100 µL of iodine-potassium iodide solution was included and, after another 10 min of incubation at 37 °C, absorbances were read at 630 nm. Acarbose was used as the standard inhibitor and the results were expressed as equivalents of acarbose (mmol ACAE/g extract) [28].

α-Glucosidase inhibitory activity was performed as per the previous method [28]. The sample solution (50 µL, 1 mg/mL) was mixed with glutathione as an antioxidant stabilizing agent for the enzyme (50 µL, 1 mg/mL), α-glucosidase solution (0.2 U/mL, from *Saccharomyces cerevisiae*, EC 3.2.1.20, Sigma, Milan, Italy) (50 µL) in phosphate buffer (pH 6.8, 0.1 mM) and 50 µL of PNPG (4-nitrophenyl-α-D-glucopyranoside, 10 mM) in a 96-well microplate for 15 min at 37 °C. Similarly, a blank was prepared by adding the sample solution to all reaction reagents without the enzyme solution. The reaction was then stopped with the addition of 50 µL of sodium carbonate solution (0.2 M). The sample and blank absorbances were read at 405 nm. The absorbance of the blank was subtracted from that of the sample and the α-glucosidase inhibitory activity was expressed as millimoles of acarbose equivalents (mmol ACAE/g extract).

Cholinesterase (ChE) inhibitory activity was measured using Ellman’s method, as previously reported [28]. The sample solution (100 µL, 1 mg/mL) was mixed with 100 µL of DTNB (5,5-dithio-bis(2-nitrobenzoic) acid, 0.3 mM) and 25 µL of AChE (acetylcholinesterase (Electric eel acetylcholinesterase, Type-VI-S, EC 3.1.1.7, Sigma, Milan, Italy), 0.026 U/mL), or BChE (butyrylcholinesterase (horse serum butyrylcholinesterase, EC 3.1.1.8, Sigma, Milan, Italy), 0.026 U/mL) solution in Tris-HCl buffer (pH 8.0, 50 mM) incubating in a 96-well microplate for 15 min at 25 °C. The reaction was then started with the addition of 25 µL of acetylthiocholine iodide (1.5 mM) or butyrylthiocholine chloride (1.5 mM). Similarly, a blank was prepared by adding the sample solution to all reaction reagents without the proper enzyme (AChE or BChE) solution. The sample and blank absorbances were read at 405 nm after a 10 min incubation at 25 °C. The absorbance of the blank was subtracted from that of the sample and the cholinesterase inhibitory activity was expressed as milligrams of galantamine equivalents (mg GALAE/g extract).

Evaluation of anti-tyrosinase potential was carried out by adding 25 µL of sample (1 mg/mL) to 40 µL of tyrosinase solution (200 U/mL) and 100 µL of phosphate buffer (40 mM, pH 6.8) in a 96-well microplate and was allowed to incubate at 25 °C for 15 min. The substrate L-DOPA (10 mM, 40 µL) was used to start the reaction. Following 10 min of incubation at room temperature, all absorbances were read at 492 nm. For all enzyme inhibition assays, a blank solution was prepared using the same respective procedures but without sample. Kojic acid was used as the positive control and results were expressed as kojic acid equivalents (mg KAE/g extract) [28].

### 3.14. Statistical Analysis

Total bioactive compounds, antioxidant, and enzyme inhibition results were expressed as means ± SD of three replications. Then, one-way ANOVA (Tukey’s assay) was performed with Xlstat 2017 software Addinsoft (Paris, France), (*p* < 0.05 was considered statistically significant) for determining differences in the extracts.

## 4. Conclusions

*Elaeagnus angustifolia* is an underused species, able to grow in a wide range of environmental conditions. The particular metabolite composition of its fruits and leaves can have positive effects on human health by reducing the onset and development of certain diseases, especially age-related ones. The results obtained gave us a better knowledge of the phytocomplex composition of the leaves and fruits of this highly adaptable plant. The different extraction methods applied enabled us to obtain the selective extraction of specific compounds or chemical classes and the multicomponent analysis allowed to show new molecules undetected before and to quantify the main components. Leaf extracts showed a higher content of TPC (confirmed by HPLC analyses) and higher antioxidant and chelating activity with respect to fruit extracts, following the trend *L_H_* ≈ *L_M_* ˃˃ *L_S_*. Both leaf and fruit extracts expressed a significant inhibition of tyrosinase, but also, in this case, leaf extracts provided a higher potential.

## Figures and Tables

**Table 1:** Colorimetric CIEL*a*b* parameters of *Elaeagnus* powders and extracts; each reported value is the mean of four measurements.

	*Lp*	*L_H_*	*L_M_*	*Fp*	*F_H_*	*F_M_*
L*	56.29 ± 1.52	40.52 ± 0.33	41.22 ± 2.44	68.61 ± 0.64	58.66 ± 2.46	58.90 ± 1.46
a*	−1.47 ± 0.49	−7.80 ± 0.49	−8.98 ± 3.26	7.73 ± 0.02	−2.15 ± 0.20	−2.30 ± 0.32
b*	13.14 ± 1.68	14.41 ± 1.72	22.41 ± 4.58	27.23 ± 0.31	8.62 ± 0.16	8.46 ± 3.07
C*	13.24 ± 1.61	16.39 ± 1.73	24.18 ± 5.45	28.31 ± 0.29	8.88 ± 0.12	8.78 ± 3.04
*h_ab_*	96.62 ± 2.96	118.57 ± 1.58	111.22 ± 3.36	74.14 ± 0.18	104.01 ± 1.46	106.29 ± 3.70

**Table 2:** Chlorophyll and carotenoid contents of *Elaeagnus angustifolia* fruits and leaves.

	Lp *	Fp *
Chlorophyll a	43.8 ± 8.6	1.0 ± 0.4
Chlorophyll b	24.0 ± 4.0	1.9 ± 0.2
Total carotenoids	18.3 ± 2.5	3.2 ± 0.6

* The data are expressed in µg/g of dry extract.

**Table 3:** Chemical composition (%) of vapor phase of dried leaves and fruits of *Elaeagnus angustifolia*.

N°	Compound ^1^	LRI ^2^	LRI^lit 3^	*F_P_* (%)	*L_P_* (%)
1	dihydro-2-methyl-furanone	773	775	5.8	-
2	pyrimidine, 2-methyl	790	^+^	-	7.2
3	furfural	798	802	36.7	18.8
4	2-furanmethanol	833	835	7.0	5.8
5	acetol acetate	858	862	0.2	3.2
6	butyrolactone	861	863	-	-
7	*p*-xylene	867	869	-	5.3
8	2(5*H*)-furanone	868	871	1.2	-
9	4-cyclopentene-1,3-dione	880	884	4.5	-
10	acetyl furan	910	914	2.5	1.8
11	pyrrolidine, 2-(cyanomethylene)	922	^+^	-	8.1
12	5-methylfurfural,	930	933	25.3	9.6
13	5-hepten-2-one, 6-methyl-	956	962	-	2.0
14	2-furanmethanol, acetate	960	966	0.4	-
15	2-ethyl-6-metylpyrazine	982	981	-	2.1
16	2,4-dihydroxy-2,5-dimethyl-3-(2*H*)-furanone	980	989°	1.0	3.2
17	2-cyclopenten-1-one, 2-hydroxy-3-methyl-	997	1000	0.7	-
18	1*H*-pyrrole-2-carboxaaldehyde	1004	1009	-	6.4
19	2,5-furandione, 3,4-dimethyl-	1032	1038	1.4	-
20	2,5-dimethyl-4-hydroxy-3(2*H*)-furanone	1062	1064	4.7	3.2
21	2-acetylpyrrole	1066	1065	0.9	4.3
22	nicotinyl acetate	1103	1100	-	-
23	pyrimidine-4,6-diol,5-methyl	1105	-	1.1	-
24	pyranone	1110	1107	1.8	-
25	5-hydroxymethylfurfural	1202	1208	2.7	-
26	*p*-vinylguiacol	1287	1282	-	9.3
27	5-acetoxymethyl-2-furaldheyde	1305	*	0.6	-
28	naphthalene,1,2-dihydro-1,1,6-trimethyl-	1328	1332°	-	3.7
29	geranylacetone	1429	1426	-	1.7
30	dehydro β-ionone	1435	1433	-	1.2
31	dihydroactinidiolid	1461	1458	-	1.4
32	hexahydrofarnesyl acetone	1850	1846	-	1.2
Total (%)				98.5	99.5

^1^ Elution order on apolar column; ^2^ Linear Retention Indices (LRI) measured on apolar column; ^3^ Linear Retention Indices from literature; ^+^ LRI only for polar column; ° LRI for Normal Alkane; * LRI not available for temperature ramp; -: Not detected.

**Table 4:** Chemical composition (%) of *E. angustifolia* extracts.

N°	Compound ^1^	LRI ^2^	LRI^lit 3^	*F_M_* (%)	*L_M_* (%)	*F_H_* (%)	*L_H_* (%)
1	Acetol	1319	1317	6.4	20.4	18.6	24.0
2	methyl pyruvate	1322	*	-	7.5	-	11.4
3	acetic acid	1440	1442	-	14.1	-	16.0
4	furfural	1458	1465	28.7	-	2.3	-
5	acetylfuran	1495	1497	2.1	-	2.9	-
6	5-methyl furfural	1600	1604	11.3	-	8.9	-
7	2-furanmethanol	1655	1659	7.7	-	18.2	-
8	ionone	1840	1846	-	10.0	-	8.9
9	furaneol	2056	2060	16.9	-	5.4	-
10	*p*-vinylguaiacol	2160	2166	-	48.0	-	39.5
11	5-acetoxymethyl-2-furaldheyde	2195	2199	4.7	-	4.7	-
12	pyranone	2271	2274	10.9	-	17.3	-
13	5-hydroxymethylfurfural	2529	2532	11.3	-	21.6	-
Total (%)				100.0	100.0	99.9	99.8

^1^ Elution order on polar column; ^2^ Linear Retention Indices measured on polar column; ^3^ Linear Retention Indices from literature; * LRI not available for temperature ramp; -: Not detected.

**Table 5:** HPLC-DAD quantitative analysis, expressed in mg/g of dry extract.

	*L_H_*	*L_M_*	*L_S_*	*F_H_*	*F_M_*	*F_S_*
Epicatechin	13.30 ± 2.60	43.10 ± 1.70	nd	nd	nd	nd
Chlorogenic acid	40.8 ± 1.52	41.9 ± 1.32	4.85 ± 0.10	1.10 ± 0.20	3.50 ± 0.80	nd
Caffeic acid	2.40 ± 0.12	3.18 ± 0.50	nd	0.19 ± 0.06	0.40 ± 0.10	nd
*p*-Coumaric acid	0.18 ± 0.06	0.20 ± 0.04	nd	nd	nd	nd
Ferulic acid	0.50 ± 0.09	0.70 ± 0.08	nd	nd	nd	nd
Flavonoids	5.40 ± 0.35	6.79 ± 0.10	0.80 ± 0.08	nd	nd	nd

The sum of the areas of the peaks identified as flavonoids is expressed as mg of quercetin-3-d-galactoside; nd: not detected.

**Table 6:** TPC, TFC, and antioxidant assays for leaf and fruit *Elaeagnus angustifolia* extracts.

Samples	TPC (mg GAE/g)	TFC (mg RE/g)	DPPH (mg TE/g)	ABTS (mg TE/g)	CUPRAC (mg TE/g)	FRAP (mg TE/g)	Metal Chelating Ability (mg EDTAE/g)	Phosphomolybdenum Assay (mmol TE/g)
*F_H_*	5.15 ± 0.03 ^d^	0.22 ± 0.02 ^d^	na	5.06 ± 0.05 ^d^	11.92 ± 0.09 ^c^	6.47 ± 0.03 ^c^	2.64 ± 0.30 ^c^	0.64 ± 0.02 ^d^
*F_M_*	4.84 ± 0.05 ^d^	0.21 ± 0.08 ^d^	na	3.62 ± 0.39 ^e^	12.34 ± 0.51 ^c^	6.51 ± 0.10 ^c^	2.43 ± 0.25 ^c^	0.56 ± 0.03 ^d^
*F_S_*	3.32 ± 0.04 ^e^	0.17 ± 0.02 ^d^	na	2.22 ± 0.21 ^f^	11.59 ± 0.07 ^c^	6.03 ± 0.04 ^c^	2.97 ± 0.16 ^c^	0.50 ± 0.02 ^d^
*L_H_*	65.35 ± 0.52 ^a^	32.91 ± 0.12 ^b^	48.93 ± 0.06^a^	72.44 ± 0.40 ^a^	86.67 ± 2.51 ^a^	45.66 ± 0.67 ^a^	11.97 ± 1.36 ^b^	2.46 ± 0.14 ^b^
*L_M_*	57.67 ± 0.09 ^b^	36.58 ± 0.35 ^a^	48.85 ± 0.01 ^a^	69.64 ± 0.38 ^b^	86.24 ± 1.85 ^a^	45.40 ± 0.86 ^a^	11.13 ± 1.14 ^b^	2.20 ± 0.08 ^c^
*L_S_*	17.86 ± 0.70 ^c^	24.34 ± 0.24 ^c^	2.57 ± 0.29 ^b^	13.18 ± 0.40 ^c^	67.03 ± 1.29 ^b^	20.33 ± 0.19 ^b^	18.25 ± 0.44 ^a^	2.73 ± 0.14 ^a^

Values are expressed as mean ± SD. na: not active. GAE: Gallic acid equivalent; RE: Rutin equivalent; EDTAE: EDTA equivalent; TE: Trolox equivalent. Different letters indicate significant differences in the extracts (*p* < 0.05).

**Table 7:** Enzymatic inhibition assays for leaf and fruit *Elaeagnus angustifolia* extracts.

Samples	AChE Inhibition (mg GALAE/g)	BChE Inhibition (mg GALAE/g)	Tyrosinase Inhibition (mg KAE/g)	α-Amylase Inhibition (mmol ACAE/g)	α-Glucosidase Inhibition (mmol ACAE/g)
*F_H_*	1.72 ± 0.08 ^c^	3.37 ± 0.31 ^b^	35.42 ± 0.75 ^c^	0.15 ± 0.01 ^e^	na
*F_M_*	2.02 ± 0.10 ^b^	3.51 ± 0.19 ^b^	37.53 ± 2.62 ^b,c^	0.22 ± 0.01 ^d^	na
*F_S_*	2.54 ± 0.01 ^a^	4.53 ± 0.10 ^a^	37.21 ± 2.35 ^b,c^	0.38 ± 0.01 ^c^	0.69 ± 0.02 ^b^
*L_H_*	2.08 ± 0.07 ^b^	1.11 ± 0.06 ^c^	61.20 ± 3.65 ^a^	0.39 ± 0.01 ^c^	0.83 ± 0.01 ^a^
*L_M_*	2.21 ± 0.04 ^b^	1.48 ± 0.13 ^c^	58.84 ± 1.02 ^a^	0.45 ± 0.01 ^b^	0.85 ± 0.01 ^a^
*L_S_*	0.88 ± 0.09 ^d^	4.81 ± 0.30 ^a^	43.21 ± 3.56 ^b^	0.60 ± 0.02 ^a^	0.65 ± 0.01 ^c^

Values are expressed as mean ± SD. GALAE: Galantamine equivalent; KAE: Kojic acid equivalent; ACAE: Acarbose equivalent; na: not active. Different letters indicate significant differences in the extracts (*p* < 0.05).

## Supplementary Materials

The following are available online at https://www.mdpi.com/1420-3049/25/9/2021/s1, Figure S1: ^1^H NMR (400.13 MHz) DMSO*-d*_6_ (*F_S_* sample); Figure S2: ^1^H NMR (400.13 MHz) DMSO*-d*_6_ (5-(hydroxymethyl)furfural); Figure S3: ^13^C NMR (100.03 MHz) DMSO*-d*_6_; Figure S4: ^1^H NMR (400.13 MHz) DMSO*-d*_6_ (4,5,6-Trihydroxyhex-3-en-2-one); Figure S5: ^13^C NMR (100.03 MHz) DMSO*-d*_6_; Figure S6: MASS SPECTRUM of 5-Hydroxymethylfurfural; Figure S7: MASS SPECTRUM of *2-*Furanmethanol; Figure S8: MASS SPECTRUM of 5-Methylfurfural Figure S9: MASS SPECTRUM of Furfural; Figure S10: MASS SPECTRUM of Acetic acid; Figure S11: MASS SPECTRUM of Acetol Figure S12: MASS SPECTRUM OF p-Vinylguaiacol; Figure S13: MASS SPECTRUM of Pyranone.
