# Supplementary material for: Phytocomplex Characterization and Biological Evaluation of Powdered Fruits and Leaves from Elaeagnus angustifolia
Source: Molecules. 2020 Apr 26;25(9):2021. doi: 10.3390/molecules25092021 (PMC7248930; doi:10.3390/molecules25092021)
Supplement: Supplementary file 1 [file molecules-25-02021-s001.pdf]

molecules

# Supplementary Materials

**Simone Carradori [1,*], Francesco Cairone [2], Stefania Garzoli [2], Giancarlo Fabrizi [2], Antonia Iazzetti [2], Anna Maria Giusti [3], Luigi Menghini [1], Sengul Uysal [4,5], Gunes Ak [6], Gokhan Zengin [6] and Stefania Cesa [2,*]**

1 Department of Pharmacy, University "G. d'Annunzio" of Chieti-Pescara, Chieti 66100, Italy; luigi.menghini@unich.it
2 Department of Drug Chemistry and Technologies, "Sapienza" University of Rome, 00185 Rome, Italy; francesco.cairone@uniroma1.it (F.C.); stefania.garzoli@uniroma1.it (S.G.); giancarlo.fabrizi@uniroma1.it (G.F.); antonia.iazzetti@uniroma1.it (A.I.)
3 Department of Experimental Medicine, "Sapienza" University of Rome, 00185 Rome, Italy; annamaria.giusti@uniroma1.it
4 Erciyes University Halil Bayraktar Health Services Vocational College, Kayseri 38039, Turkey; sennguluysal@gmail.com
5 Ziya Eren Drug Application and Research Center, Erciyes University, Kayseri 38039, Turkey;
6 Department of Biology, Science Faculty, Selcuk University, Konya 42130, Turkey; akguneselcuk@gmail.com (G.A.); gokhanzengin@selcuk.edu.tr (G.Z.)
* Correspondence: simone.carradori@unich.it (Simone Carradori) and stefania.cesa@uniroma1.it (Stefania Cesa)

Academic Editor: Ericsson Coy-Barrera
Received: 25 March 2020; Accepted: 22 April 2020; Published: date

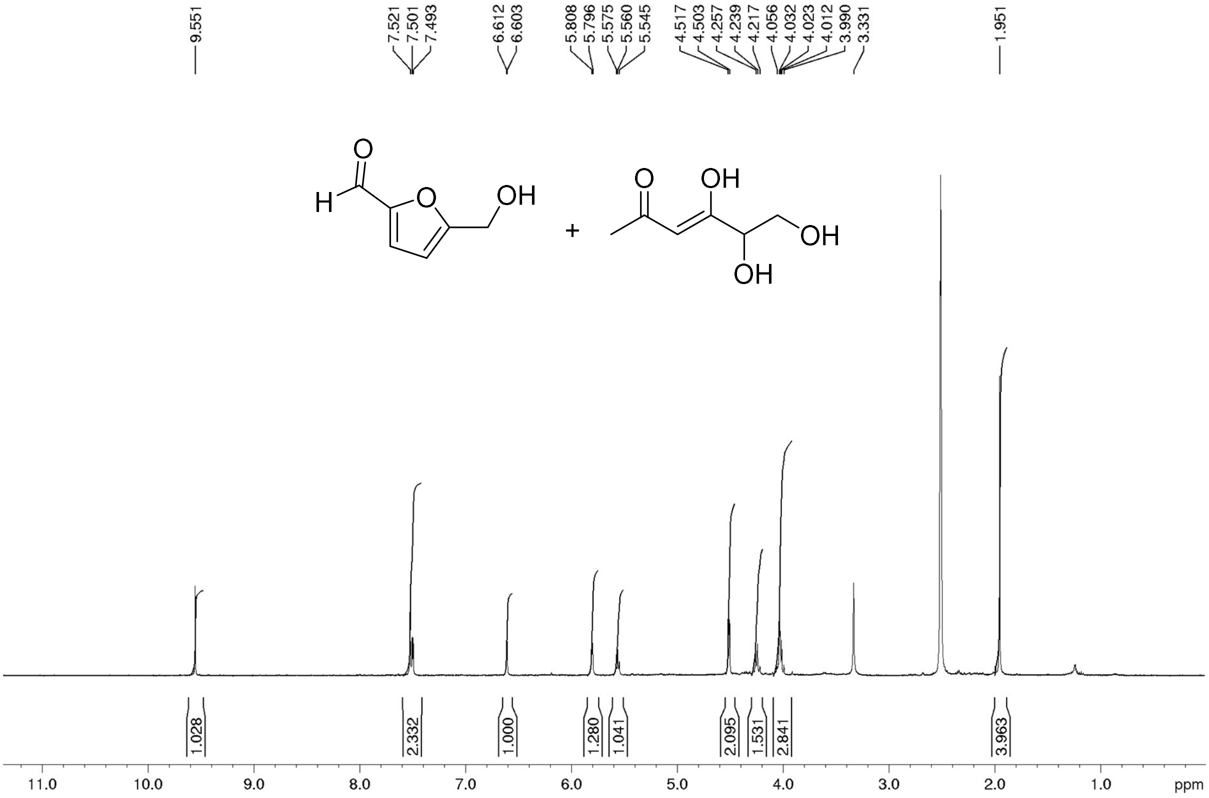


**Figure S1.** $^{1}H$ NMR (400.13 MHz) DMSO-$d_6$ ($F_5$ sample).

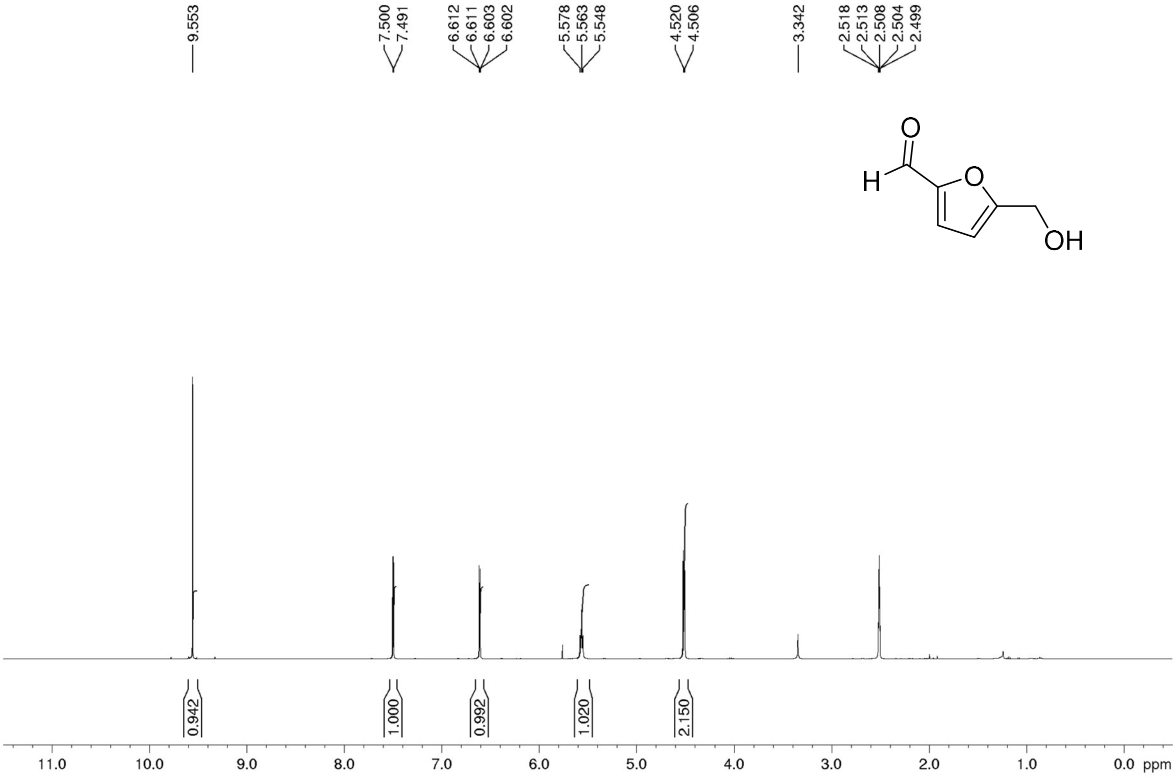


**Figure S2.** $^{1}H$ NMR (400.13 MHz) DMSO-$d_6$ (5-(hydroxymethyl)furfural).

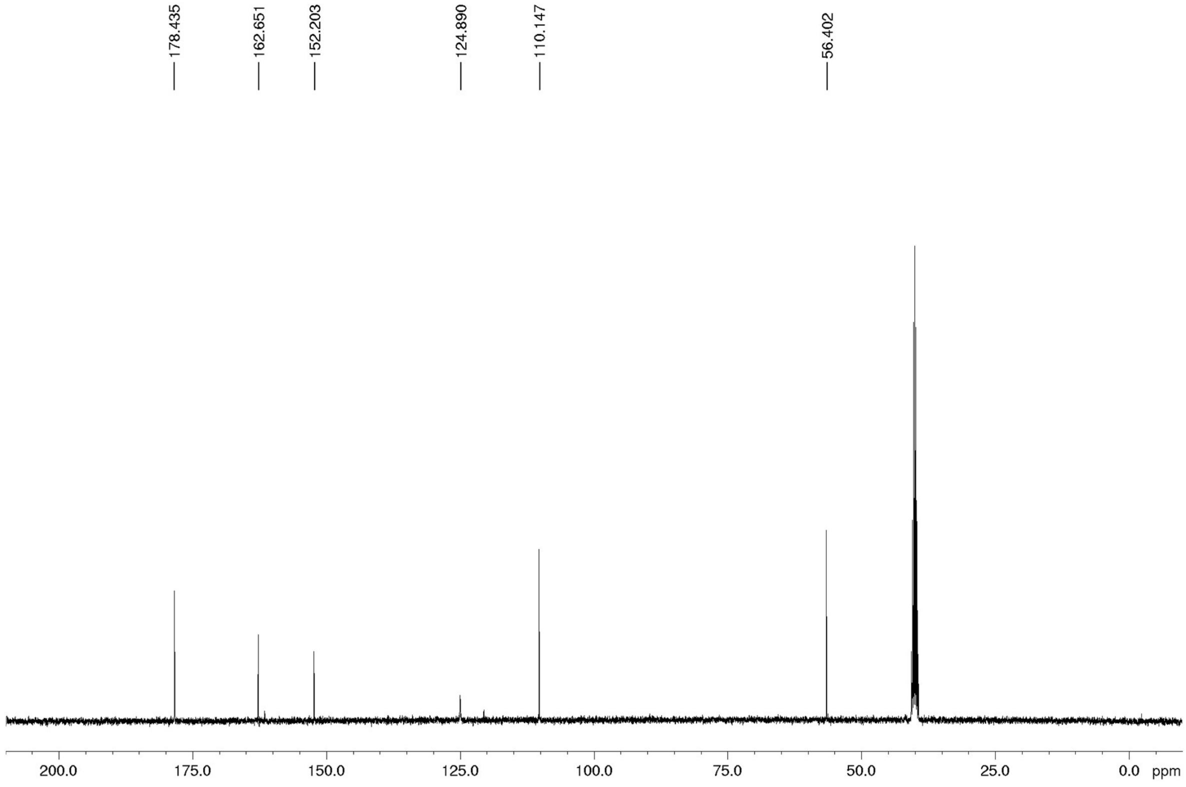


**Figure S3.** $^{13}C$ NMR (100.03 MHz) DMSO-$d_6$.

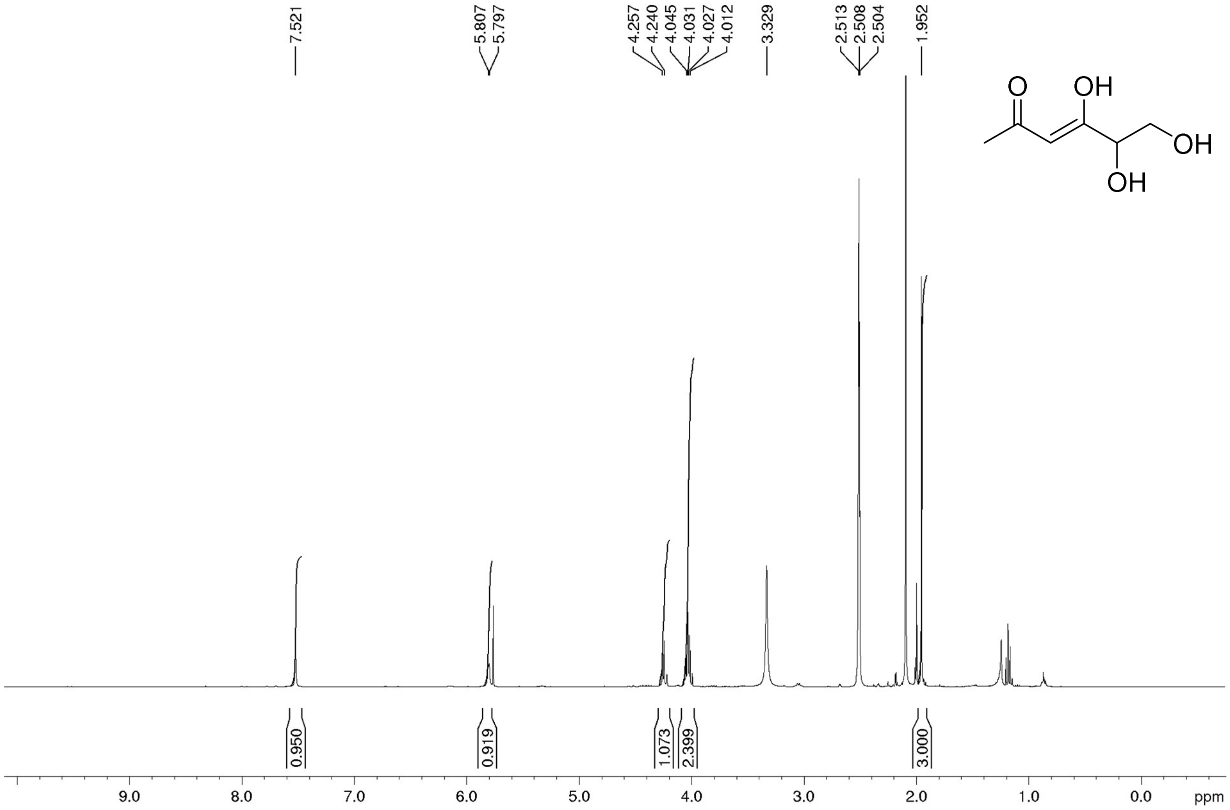


**Figure S4.** $^{1}H$ NMR (400.13 MHz) DMSO-$d_6$ (4,5,6-Trihydroxyhex-3-en-2-one).

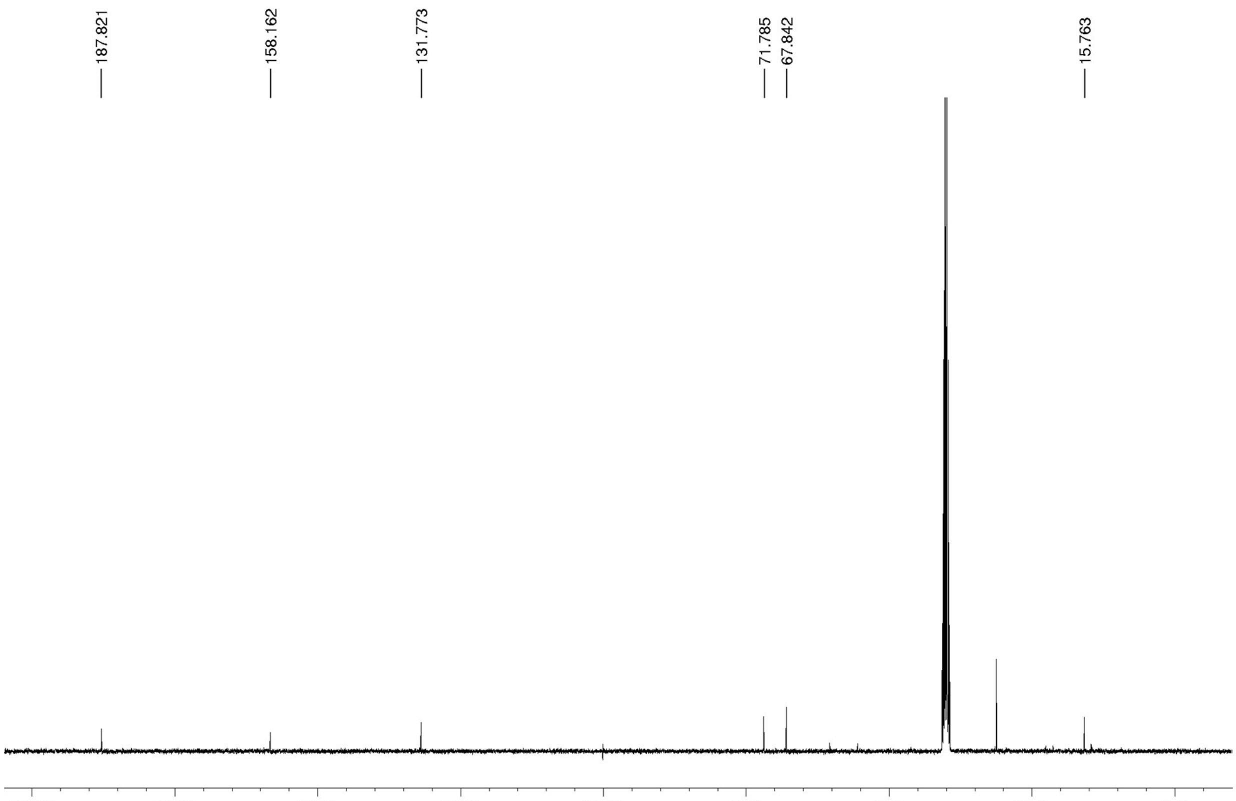


**Figure S5.** $^{13}C$ NMR (100.03 MHz) DMSO-$d_6$.

**Figure S6.** MASS SPECTRUM OF *5-HYDROXYMETHYLFURFURAL.*

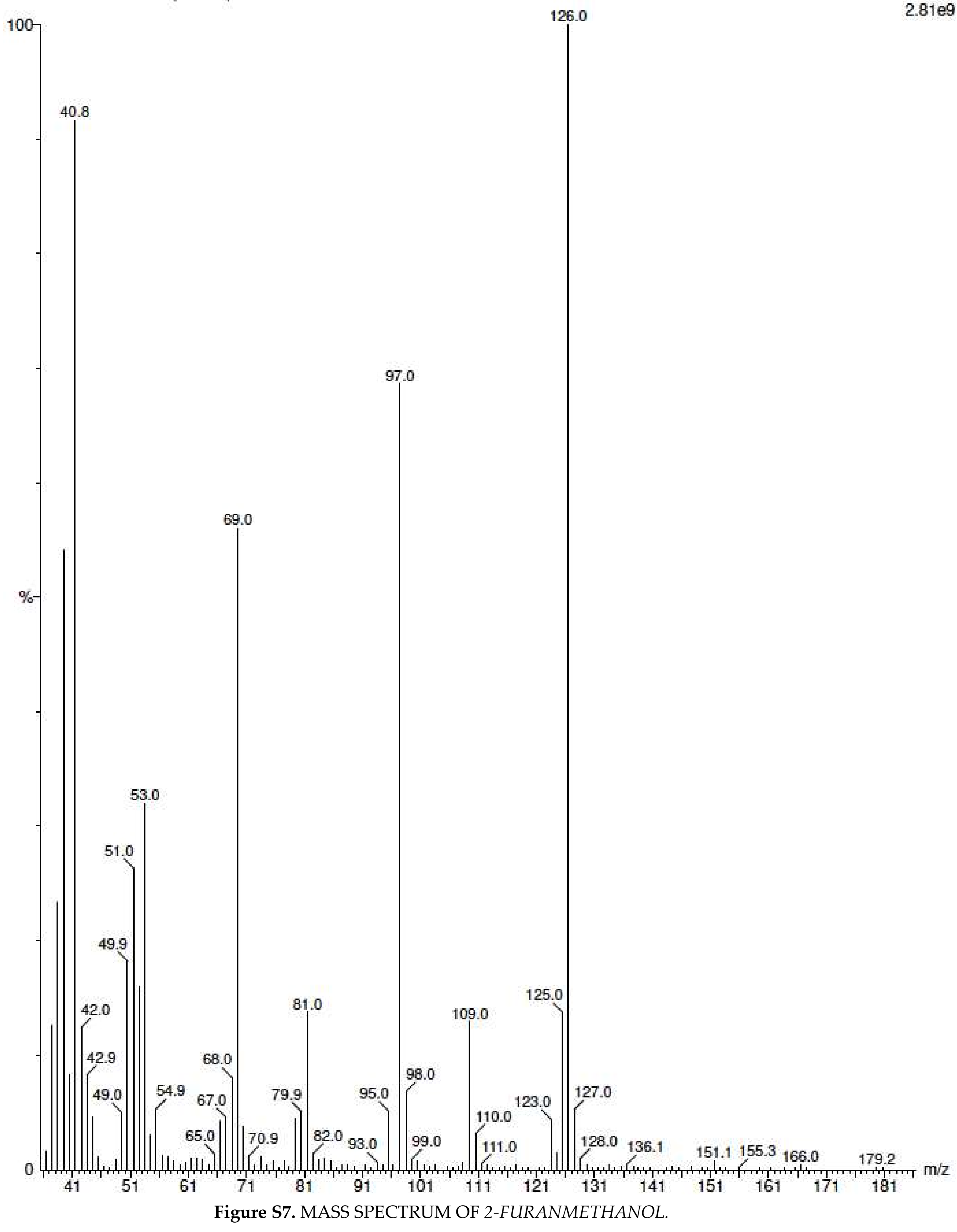


**Figure S7.** MASS SPECTRUM OF *2-FURANMETHANOL.*

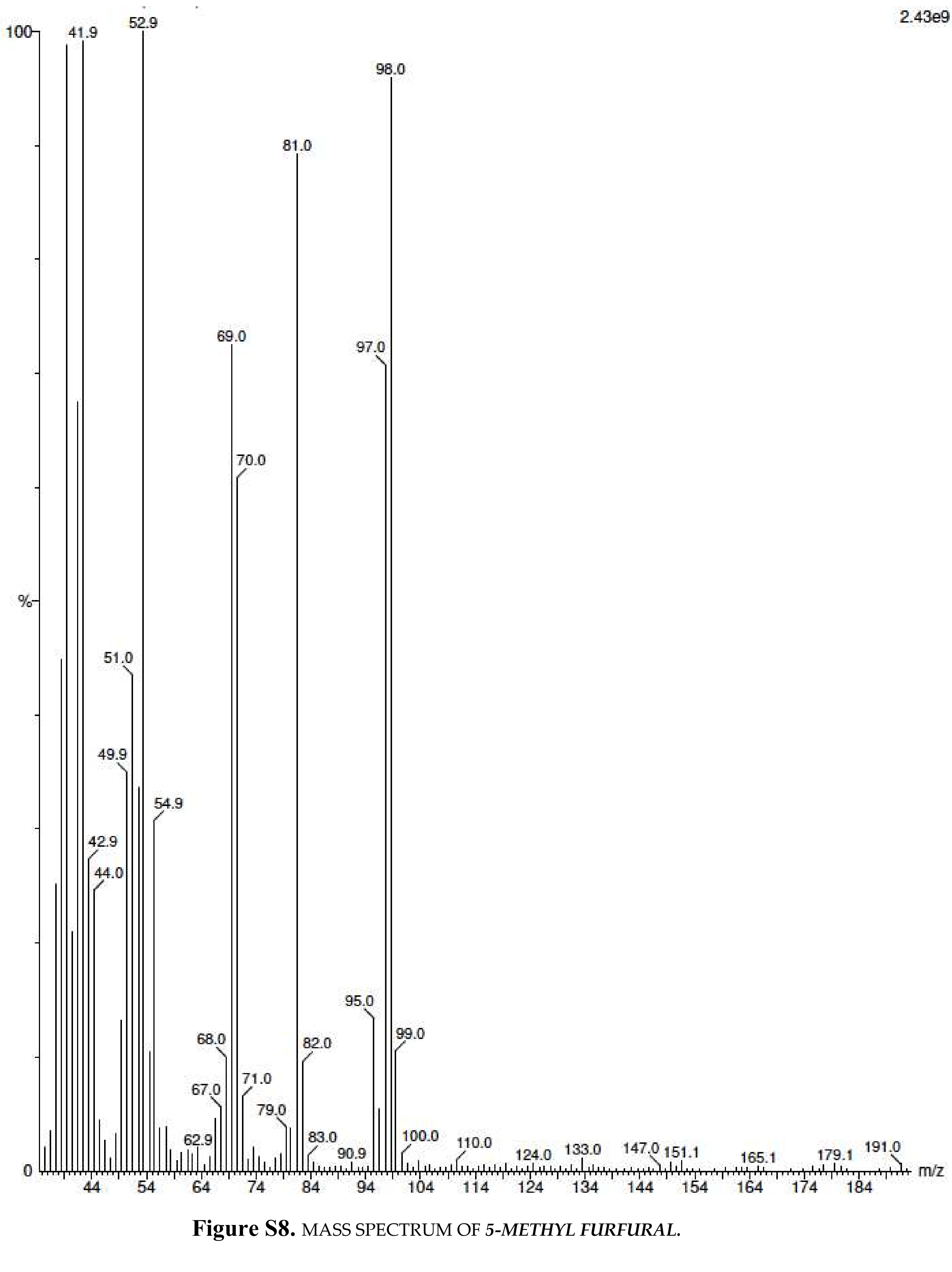


**Figure S8.** MASS SPECTRUM OF ***5-METHYL FURFURAL.***

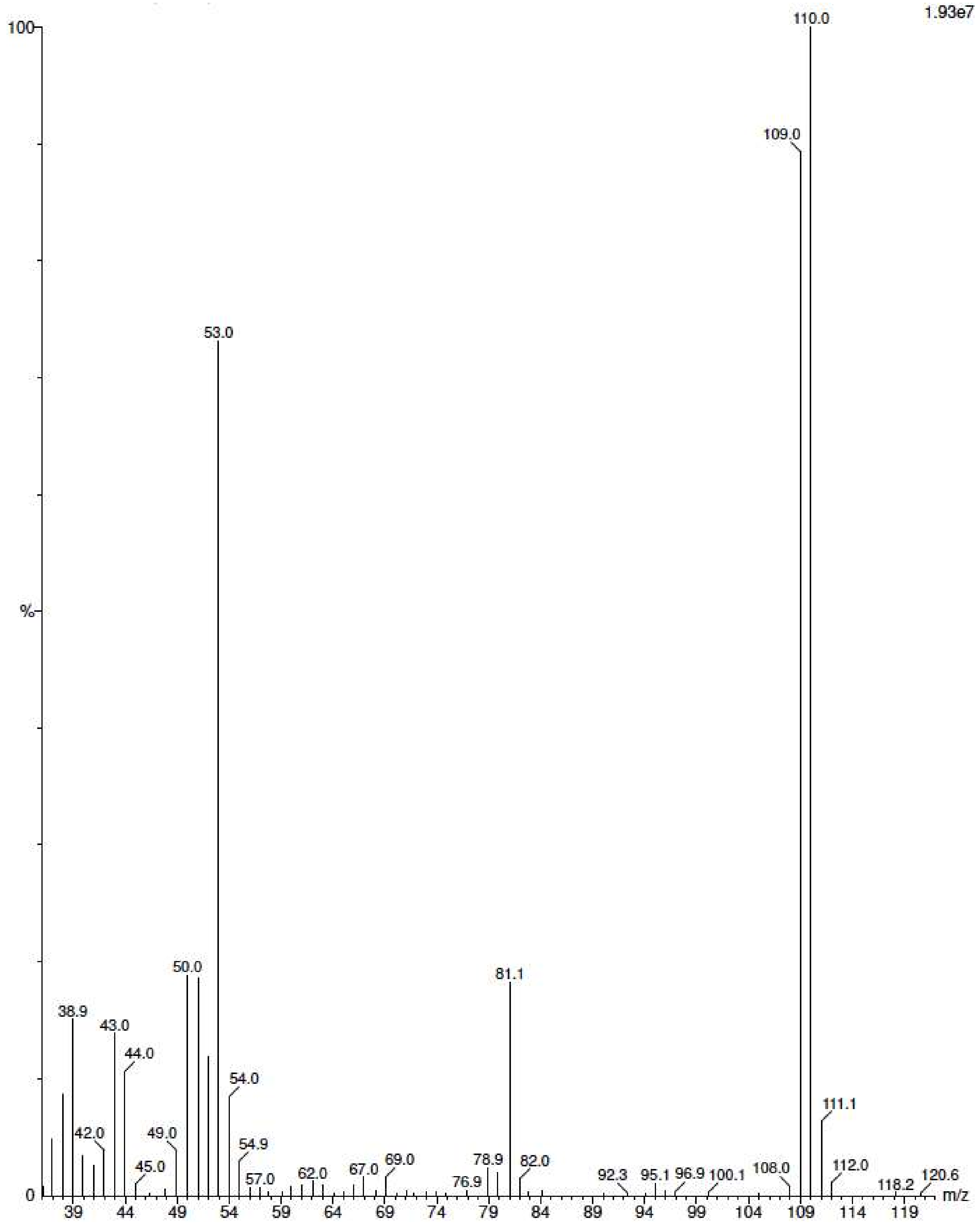


**Figure S9.** MASS SPECTRUM OF ***FURFURAL***.

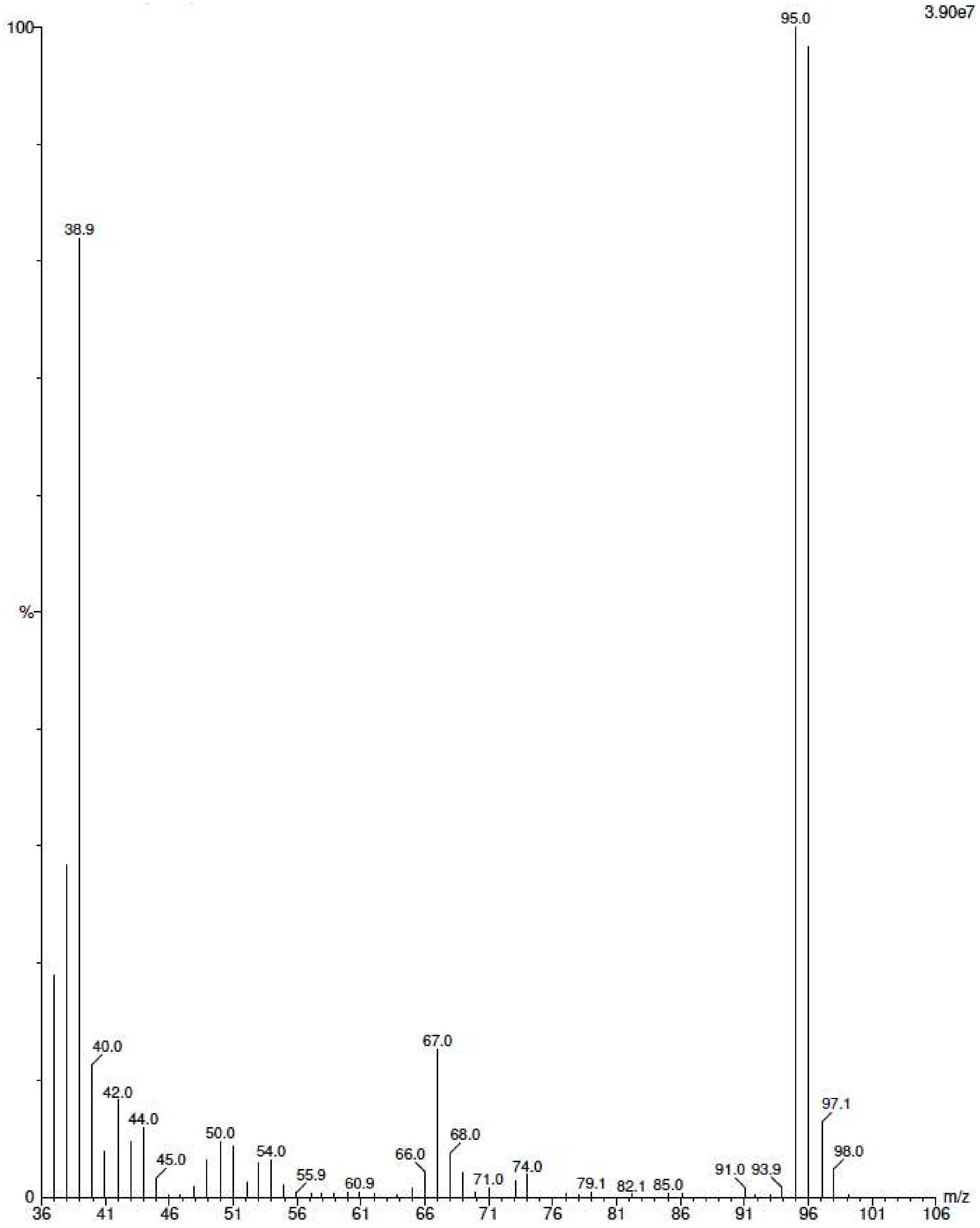


**Figure S10.** MASS SPECTRUM OF *ACETIC ACID.*

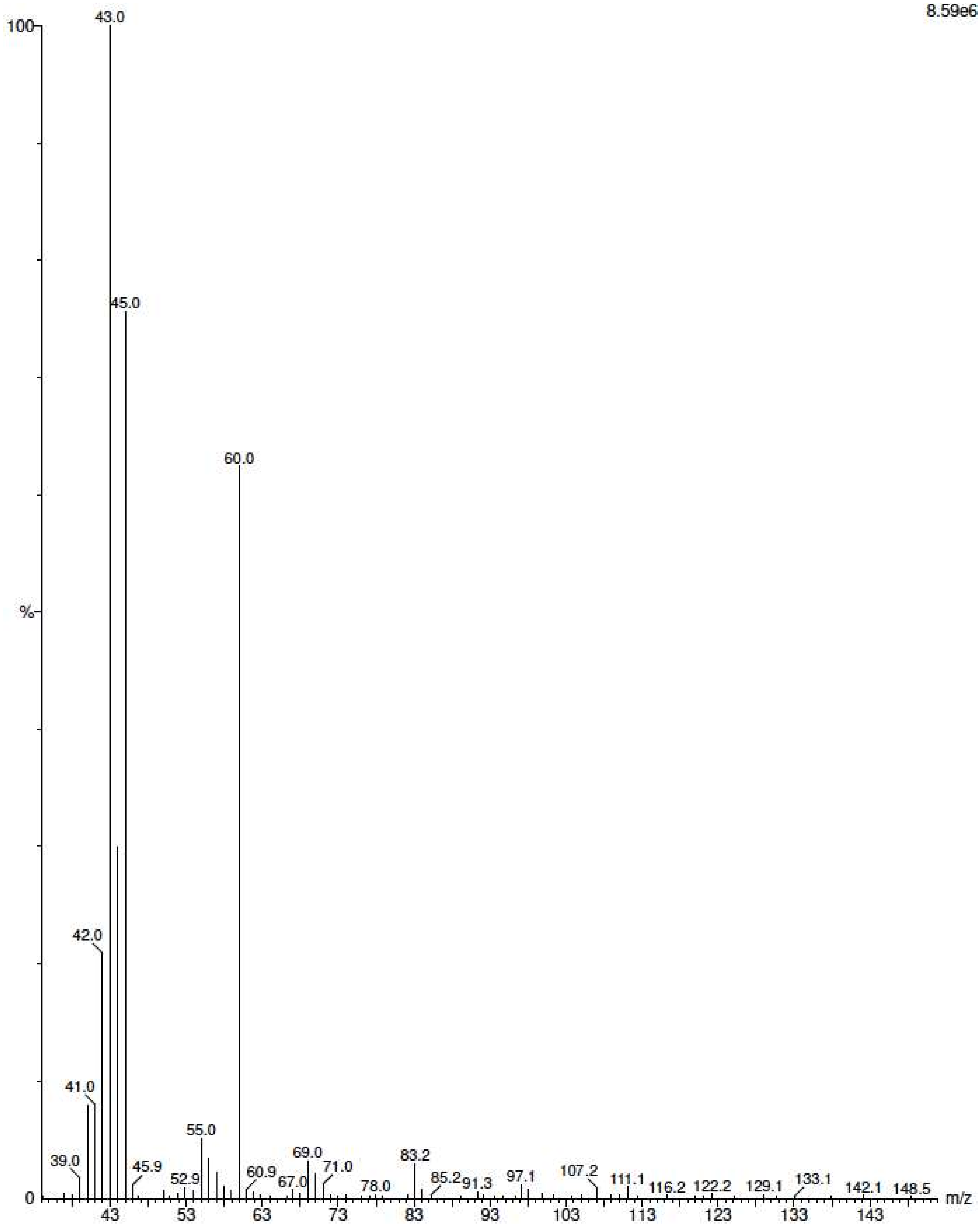


**Figure S11.** MASS SPECTRUM OF *ACETOL*.

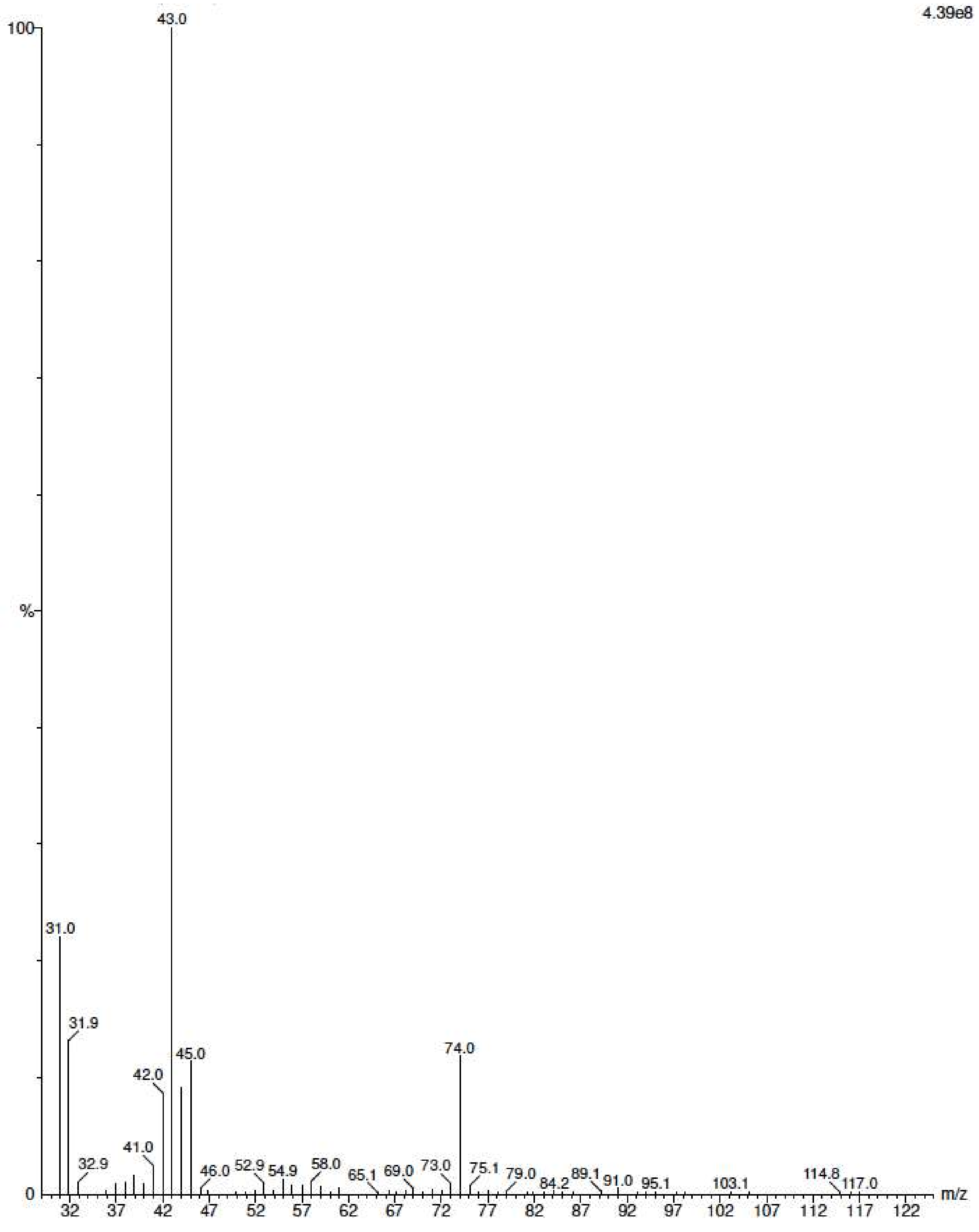


**Figure S12.** MASS SPECTRUM OF ***P-VINYLGUAIACOL***.

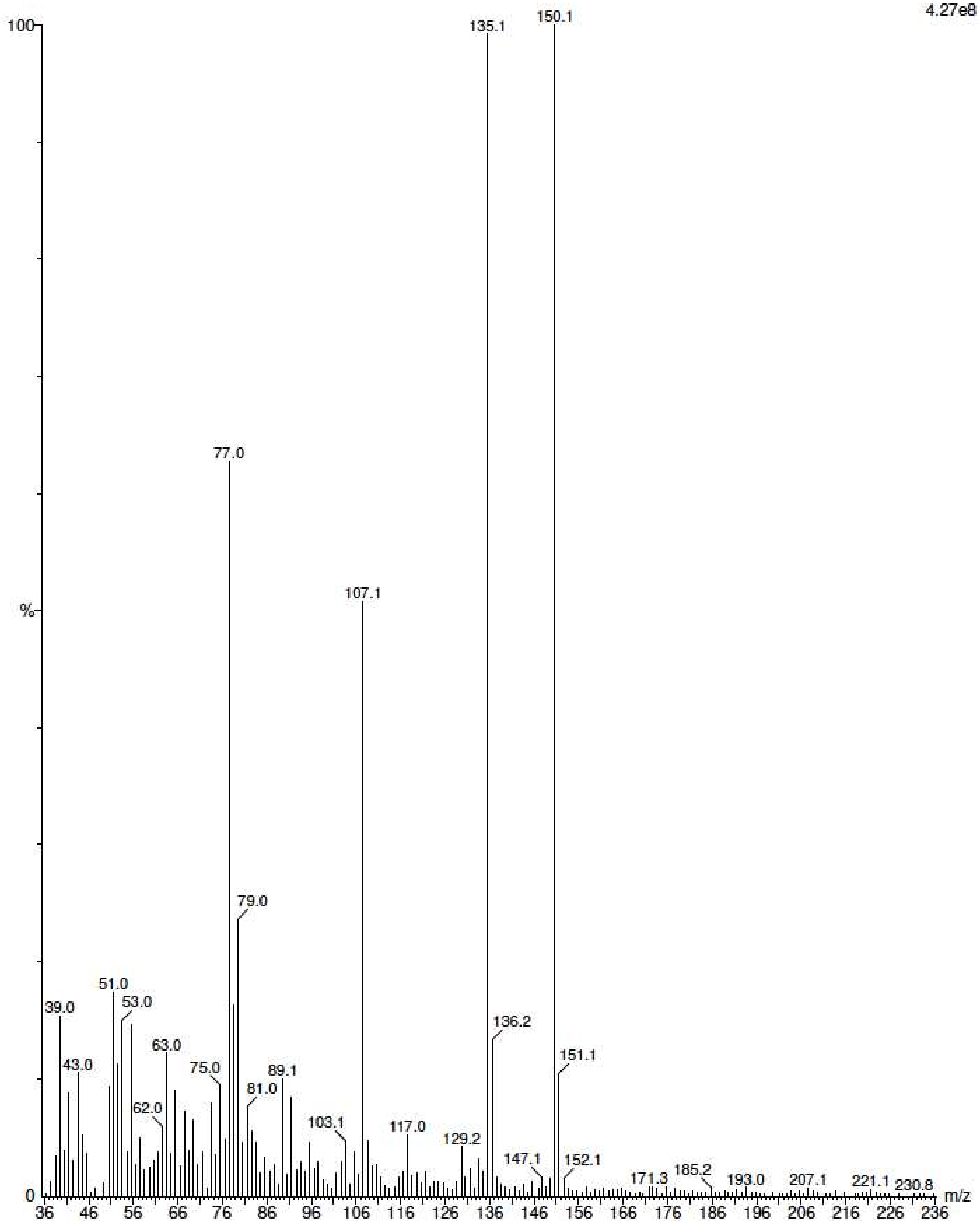


**Figure S13.** MASS SPECTRUM OF ***PYRANONE.***

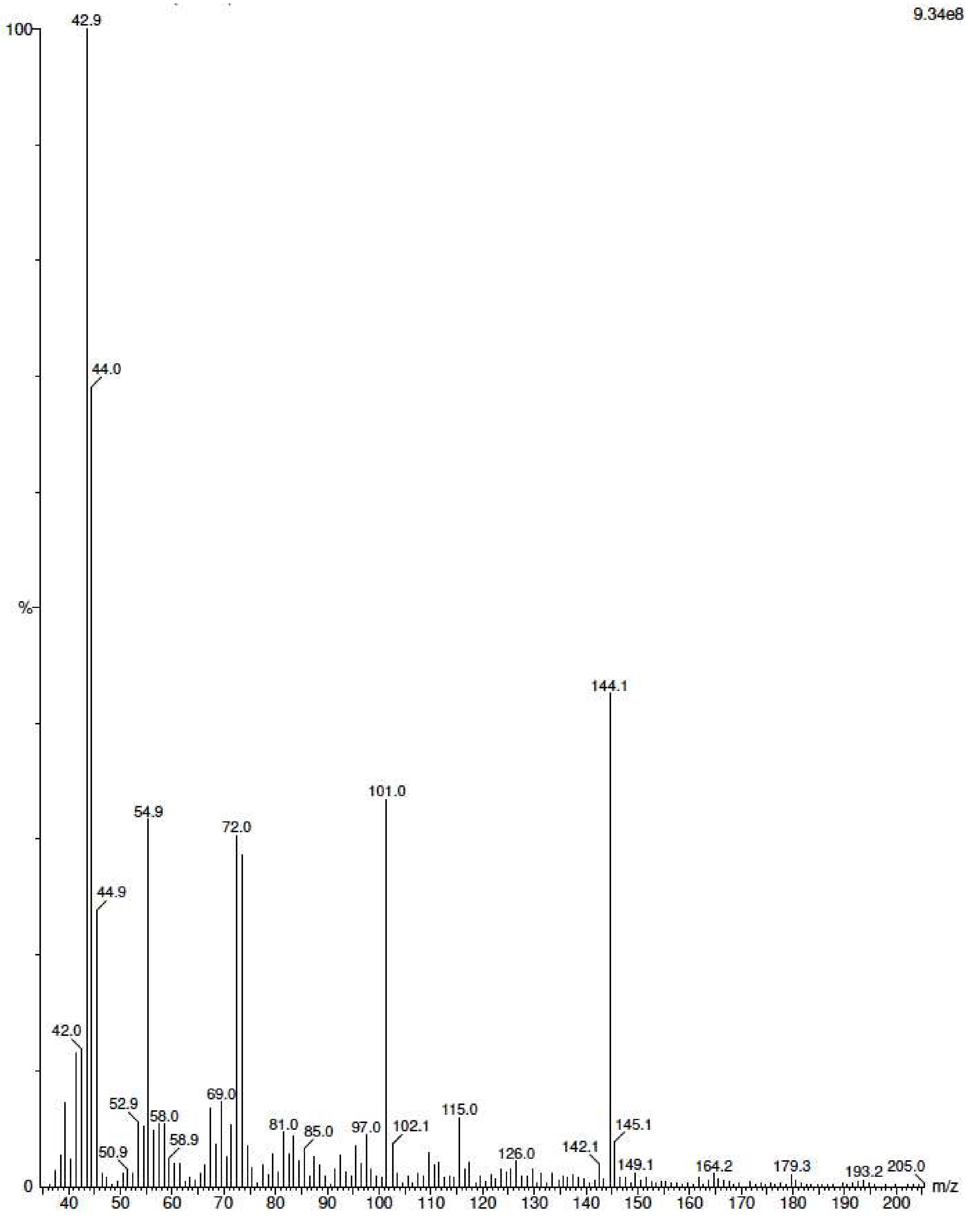
9.34e8
100
%
0
42.9
44.0
44.9
42.0
54.9
52.9
50.9
58.0
58.9
69.0
72.0
81.0
85.0
97.0
101.0
102.1
115.0
126.0
142.1
144.1
145.1
149.1
164.2
179.3
193.2
205.0
40
50
60
70
80
90
100
110
120
130
140
150
160
170
180
190
200
m/z

Figure S14
